# Computation of Nonradiative Rates for High-Throughput Virtual Screening: Application to the Discovery of Potential TADF Molecules

**DOI:** 10.1021/acs.jctc.6c00881

**Published:** 2026-08-20

**Authors:** Teodoro Pizza, Alessandro Troisi, Amedeo Capobianco

**Affiliations:** † Dipartimento di Chimica e Biologia “A. Zambelli”, 19028 Università di Salerno, Via Giovanni Paolo II, 132, I-84084 Fisciano, SA, Italy; ‡ Department of Chemistry and Materials Innovation Factory, University of Liverpool, Liverpool, L69 7ZD, U.K.

## Abstract

High-throughput virtual screening of molecules with suitable electronic properties rarely includes the computation of photophysical rate constants as target quantities. In this work, we develop a fully automated screening workflow capable of computing reverse intersystem crossing (RISC) rates for a large set of systems. The workflow is specifically designed to identify promising thermally activated delayed fluorescence (TADF) emitters and is applied to a medium-sized data set of 70 molecules. These candidates were selected from a database of 150,000 conjugated organic molecules not specifically tailored for TADF, based on desirable excited-state properties at the ground-state geometry (vertical singlet–triplet energy gap and oscillator strength). RISC rates were computed using a fully quantum-mechanical approach based on Fermi’s golden rule, which explicitly accounts for all nuclear degrees of freedom. The workflow autonomously performs the initial screening steps by excluding molecules that fail the required geometry optimization and frequency calculations and by diagnosing cases that cannot be reliably treated within the proposed framework, thereby ensuring robustness and reliability. Our results show that including RISC rate calculations drastically reduces the number of viable TADF candidates. This reduction arises from the combined effect of the key parameters governing the rate, mainly the adiabatic singlet–triplet energy gap and the reorganization energy.

## Graphical abstract

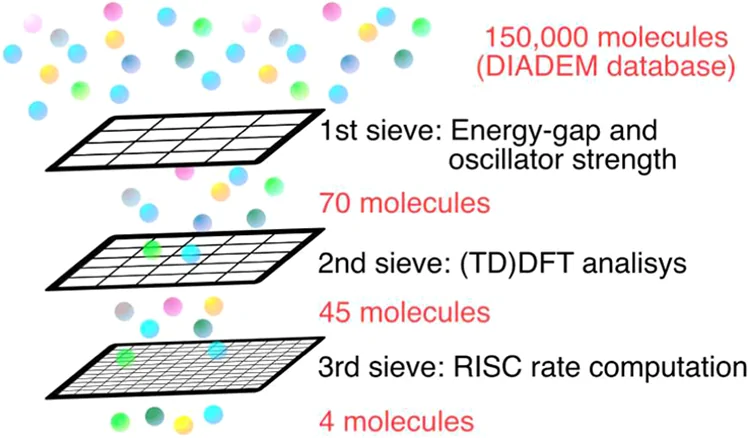

## Introduction

Over the last two decades, organic light-emitting diodes (OLEDs) have emerged as promising alternatives to their inorganic counterparts, capturing a significant share of the light-emitting diode market owing to several intrinsic advantages over inorganic technologies.^1^ To achieve efficient electroluminescence, OLED devices must exhibit an internal quantum efficiency as close as possible to 100%.^2^ To this end, several strategies have been explored, among which the most recent and impactful innovation is represented by thermally activated delayed fluorescence (TADF)-based devices.^3–6^ Unlike conventional fluorescent OLEDs, TADF-based devices exploit both singlet and triplet excitons while avoiding the use of heavy metals required in phosphorescent OLEDs.^7^ In TADF emitters, radiative emission occurs via two distinct pathways: prompt and delayed fluorescence.^8^ Prompt fluorescence arises from the direct radiative decay of the first excited singlet state (S_1_), whereas delayed fluorescence originates from the upconversion of nonemissive triplet excitons into emissive singlet states through reverse intersystem crossing (RISC), i.e., the nonradiative transition from the first excited triplet state (T_1_) to S_1_.

Since the seminal work of Adachi et al.,^8^ where one of the first highly efficient TADF-based OLED was reported, substantial research efforts have been devoted to engineering the energetic landscape of these systems, particularly by designing molecules with reduced singlet–triplet energy gaps, Δ*E*_ST_ = *E*_S_1__ – *E*_T_1__.^9–13^ A small energy gap is known to enhance the efficiency of the upconversion process and, together with a non-negligible oscillator strength for the S_1_ → S_0_ transition, to improve delayed fluorescence emission. Small Δ*E*_ST_ values are usually achieved by designing molecules with a twisted donor–acceptor architecture.^14–16^ This structural feature reduces the spatial overlap between the highest occupied and lowest unoccupied molecular orbitals (HOMO and LUMO) and, consequently, lowers the exchange energy, thereby decreasing the energy difference between the S_1_ and T_1_ excited states.

Due to its critical role in determining the overall efficiency of the TADF process, the quantitative computation of the RISC rate has been the focus of many studies.^17–23^ However, in the literature, the RISC rate is often computed at a semiclassical level and primarily for well-established TADF emitters used as reference systems, rather than as part of a systematic screening protocol.^17,19–21,24^ Furthermore, because of the complexity involved in automating the computation of rate constants, such studies are typically limited to relatively small data sets. Virtual screening approaches,^25^ have been extremely successful in identifying a large number of TADF candidates from existing experimental libraries,^11,26^ virtual libraries,^27,28^ often supported by machine learning methods^29,30^ or cheminformatics,^31^ easily yielding hundreds of proposed candidates, i.e., too many for direct experimental verification. At the same time, several recent examples clearly demonstrate the limitation of digital discovery protocols that ignore the role of nonradiative rates.^32,33^

The goal of the present work is to incorporate an accurate evaluation of the RISC rate, the key parameter governing the TADF process, in a fully automated high-throughput virtual screening (HTVS) aimed at identifying novel TADF emitters. After an initial screening layer, which isolates potential candidates from a starting database of 150,000 molecules using increasingly stringent criteria based on (TD)­DFT calculations, a fully quantum-mechanical methodology based on Fermi’s golden rule (FGR) for the computation of RISC rates is adopted. Although it has already been proven that our FGR-based methodology can provide reasonably accurate rate predictions—consistent with available experimental data^34–36^ —the broader objective of the present work is to establish a robust framework applicable to large data sets of molecules. Here, robustness refers not only to the reliability of the predicted results but also to the ability of the workflow to flag situations where the methodology may fail.

## Methods

As a preliminary step, we selected a set of potential TADF emitter molecules based solely on the vertical excitation energies and the oscillator strength of the S_1_ ← S_0_ transition. These quantities were extracted from the DIADEM database, which is derived from the ZINC database and includes more than 150,000 commercially available molecules.^37,38^ The data set adopted here was generated by selecting systems with distinct π-conjugation patterns, whose ground-state geometries were first optimized at the BLYP35/3-21G* level and subsequently subjected to single-point TDDFT calculations at the M06-2X/3-21G* level in vacuum. Details on the generation of the data set, as well as comparisons with higher levels of theory and with experimental data, are provided in the Supporting Information of ref 39. A subset of 70 molecules was selected by imposing two criteria: a vertical singlet–triplet energy gap (S_1_–T_1_ at the S_0_ equilibrium geometry) Δ*E*_ST_ lower than 0.5 eV and an oscillator strength for the vertical S_1_ ← S_0_ transition greater than 0.01. A small Δ*E*_ST_ and an appreciable oscillator strength represent necessary, although not sufficient, prerequisites for the observation of TADF. Indeed, an efficient TADF emitter is expected to feature a small Δ*E*_ST_, which favors the RISC process, and a significant transition electric dipole moment, thereby enabling both prompt and delayed fluorescence. We deliberately adopted a generous energy-gap threshold (0.5 eV) because (i) the singlet–triplet energy gap may change upon optimization of the S_1_ and T_1_ states and (ii) previous studies have reported experimentally realized TADF emitters with vertical Δ*E*_ST_ values close to 0.5 eV (Table 1 in ref 11). Importantly, this initial screening criterion did not increase the overall computational cost of the workflow since these values were already available in the DIADEM database. The list of the selected molecules and their structures are provided in Figure S1 of the Supporting Information, where all of the considered molecules are labeled with the corresponding ZINC ID.

For rate constant prediction, we employed a fully quantum-mechanical approach based on Fermi’s golden rule, following a form similar to that used in other recent studies.^23,40,41^ Because Fermi’s golden rule is derived from first-order time-dependent perturbation theory, it is valid under the condition–satisfied here–that the energy gap is much larger than the electronic coupling, which is further assumed to be independent of the nuclear coordinates (Condon approximation). Furthermore, since the RISC process is endoergic, we actually compute the nonradiative decay rate for the S_1_ → T_1_ intersystem crossing, *k*_ISC_, with this fully quantum-mechanical method, and then the RISC rate is obtained by applying the principle of detailed balance^42,43^

1 $$k_{\mathrm{RISC}}=k_{\mathrm{ISC}}e^{-\Delta E_{\mathrm{ST}}/k_{B}T}$$

where *k*_B_ is the Boltzmann constant and *T* is the temperature, which is set to 300 K in this work because we are looking for molecules with potential applications in TADF OLEDs, which usually operate at room temperature. Notably, detailed balance does not presuppose thermodynamic equilibrium; it follows from the time-reversal invariance of the quantum-mechanical Hamiltonian and therefore constrains the microscopic transition rates themselves rather than the populations of the states.^43^

Under these conditions, the intersystem crossing rate *k*_ISC_ is given by

2 $$k_{\mathrm{ISC}}=\frac{2\pi }{3\hbar }{\left|H_{\mathrm{SO}}\right|}^{2}F\left(0,T\right)$$

where *H*_SO_ is the spin–orbit coupling (expressed in cm^–1^) and is given by

$$H_{\mathrm{SO}}=\sqrt{\underset{\alpha }{\sum }{\left|H_{\mathrm{SO},\alpha }\right|}^{2}}$$

where α labels the three sublevels of T_1_.

*F*(η – Δ*E*_ST_,*T*) is the Franck–Condon weighted density of states and is given as

3 $$F\left(\eta -\Delta E_{\mathrm{ST}},T\right)=\sum_{\nu_{S_{1}\;}\;}\sum_{\nu_{T_{1}\;}\;}{\left|\left\langle \nu_{T_{1}}|\nu_{S_{1}}\right\rangle \right|}^{2}P\left(E_{\nu_{S_{1}}}\right)\delta \left(E_{\nu_{T_{1}}}-E_{\nu_{S_{1}}}-\Delta E_{\mathrm{ST}}+\eta \right)$$

where

4 $$P\left(E_{\nu_{S_{1}}}\right)=\frac{e^{-E_{\nu_{S_{1}}}/k_{B}T}}{Z_{S_{1}}}$$

is the probability of the vibrational state with energy *E*_νS_1__ to be occupied, and the independent variable η is

5 $$\eta =\{\begin{array}{ll} \pm \hbar \omega & \mathrm{radiative}\;\mathrm{transition}\\ \Delta E_{\mathrm{ST}} & \mathrm{nonradiative}\;\mathrm{transition} \end{array}$$

where the (±) sign represents emission­(absorption), *E*_νT_1__ (*E*_νS_1__) is the vibrational energy of T_1_ (S_1_), *Z*_S_1__ is the vibrational partition function of S_1_, and ⟨ν_T_1__|ν_S_1__⟩ is a Franck–Condon integral. Since nonradiative decays occur under isoenergetic conditions, the changes in vibrational energy exactly compensate for the electronic energy gap. Consequently, the FCWD is evaluated at η = Δ*E*_ST_, which corresponds to *F*(0, *T*) in eq 2. Although the FCWD formally appears as a double sum over an infinite number of states, following the seminal work of Lax and Kubo, who introduced the so-called generating function (GF) formalism, its evaluation can be recast as a discrete inverse Fourier transform of an autocorrelation function *f*(*τ*, *T*)^44,45^

6 $$F\left(\eta -\Delta E_{\mathrm{ST}},T\right)=\frac{1}{2\pi }\int_{-\infty }^{+\infty }e^{i\left(\eta -\Delta E_{\mathrm{ST}}\right)\tau }f\left(\tau ,T\right)d\tau$$

where

7 $$f\left(\tau ,T\right)=\frac{\mathrm{Tr}\left[e^{i\tau H_{T_{1}}}e^{-\left(\beta +i\tau \right)H_{S_{1}}}\right]}{\mathrm{Tr}\left[e^{-\beta H_{S_{1}}}\right]}$$

β = 1/(*k*_B_ *T*) and $H_{S_{1}}$ ( $H_{T_{1}}$ ) is the vibrational Hamiltonian operator for the S_1_ (T_1_) state. Our group recently extended this approach by introducing the use of normal coordinates and the Duschinsky affine transformation^46^

$$Q_{T_{1}}={\mathrm{JQ}}_{S_{1}}+K$$

The latter, which expresses the normal coordinates of one state (**Q**_T_1__) in terms of those of the other (**Q**_S_1__), involves computing the Duschinsky matrix (**J**), which accounts for mode-mixing effects, and the displacement vector (**K**), which describes the geometric shift between the equilibrium structures of the two electronic states. Notably, introducing the further approximation of neglecting mode mixing, i.e., assuming that the **J** matrix is equal to the identity matrix, yields only a negligible reduction in the overall computational cost of an HTVS workflow. Moreover, the importance of the Duschinsky effect cannot be assessed *a priori*, and several well-known cases have shown that mode mixing can play a significant role in ISC/RISC processes.^23,47–49^ The GF approach has already been shown to yield reliable FCWDs and rate constants.^23,34,35,40,41,50^ In particular, the robustness of our quantum-mechanical approach for rate calculations has already been established in ref 23, where it was systematically benchmarked against experimental RISC rates, where available, as well as against experimental absorption and emission FCWDs, for a representative set of TADF emitters, yielding excellent quantitative agreement.

The computational workflow for calculating the rate is illustrated in Figure 1. This workflow is designed to handle potential errors and to discard molecules or flag cases that require further specialized investigation, which lie outside the scope of the present work. All steps discussed in the remainder of this section have been fully automated by Python scripts that parse Gaussian outputs, generate new inputs, collect molecule identifiers, and write reports. The computation of relevant rates requires equilibrium geometries and Hessian matrices for the S_0_, S_1_, and T_1_ electronic states.

**Figure 1:**
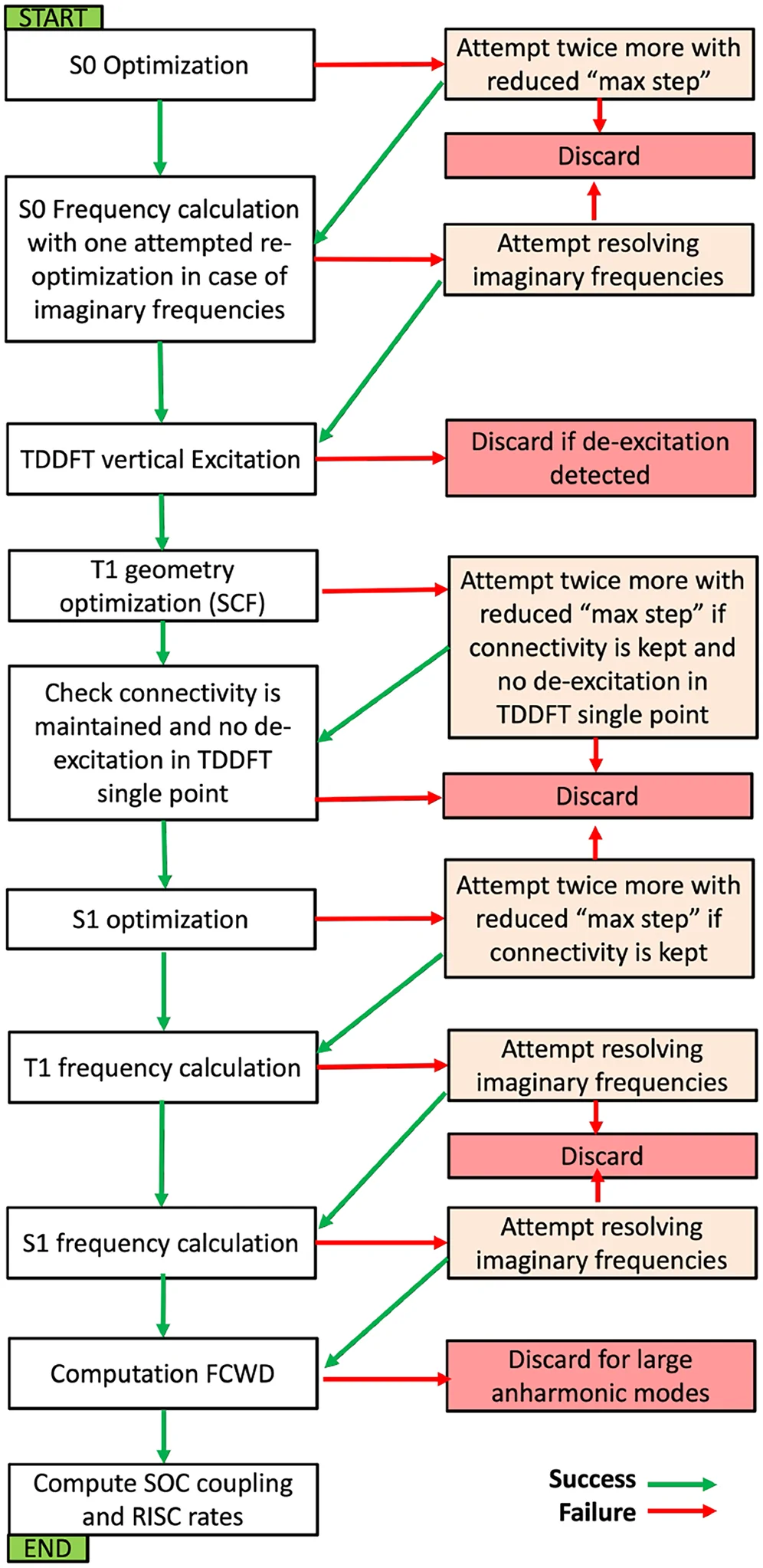
Schematic overview of the workflow developed and applied in this study.

DFT calculations were performed at the M06-2X/6-31G** level, including dielectric effects through the polarizable continuum model (PCM)^51^ with a static dielectric constant of 4.0 (a suitable value for mimicking the dielectric environment in organic solid-state devices^52^), as implemented in Gaussian.^53^ This choice is motivated by the fact that, according to eq 1, the accurate evaluation of RISC rates critically depends on reliable singlet–triplet energy gaps. In this regard, it is well established that TDDFT tends to overestimate the singlet–triplet energy gap, particularly when low-exchange functionals are employed. These functionals often fail to properly describe excited states with significant charge-transfer character,^54,55^ partly due to self-interaction error.^56^ In contrast, high-exchange functionals, such as M06-2X, generally exhibit smaller errors.^57^ Even smaller errors can be achieved using wave function-based methods, such as ADC(2) or CC2; however, due to their computational cost, they could only be used for the best candidates, and even in that case, their cost should be compared with that of directly performing experimental studies on those molecules.

As a first step, ground-state geometry optimizations, followed by frequency calculations, are performed for each molecule using the coordinates stored in the DIADEM database, obtained at the BLYP35/3-21G* level as initial guesses. Molecules for which these calculations failed are discarded. These cases include, for example, a lack of convergence during geometry optimization or persistence of imaginary frequencies in the vibrational analysis. A flag records the reason for excluding a molecule from further consideration.

Next, the protocol performs vertical TDDFT calculations of the T_1_ and S_1_ excitation energies at the ground-state equilibrium geometry. TDDFT calculations (also used for T_1_) are used to assess the nature of the electronic transitions. If de-excitation contributions are detected in the TDDFT expansion coefficients, corresponding to backward transitions from higher- to lower-energy orbitals, the system is excluded and flagged accordingly. The criterion based on de-excitation contributions is adopted because, as discussed in the literature,^58^ the occurrence of de-excitation character in TDDFT response vectors can indicate partial diradical character in the S_0_ ground state. This, in turn, may point to an underlying multireference character in excited states, for which a single-determinant description, including standard TDDFT, may become unreliable. Proper treatment of these cases would require multireference approaches (e.g., MRCI, CAS, CAS-PT2), which would lead to a prohibitive increase in the computational cost of the protocol. In addition, if either the S_0_–T_1_ or S_0_–S_1_ vertical excitation energy is below 1.7 eV, the system is flagged. A threshold of 1.7 eV was adopted, as it represents the minimum S_0_–T_1_ vertical excitation energy computed within the current data set. This criterion was established after observing that lower vertical energies often correlate with negative excitation energies during subsequent optimization steps. Notably, this criterion is used solely to highlight potential instabilities in a molecule and does not result in its automatic exclusion from the workflow.

T_1_ geometry optimization is carried out prior to the S_1_ geometry optimization since the T_1_ equilibrium geometry, obtained via ΔSCF, avoids the more computationally demanding TDDFT optimization required for S_1_. As a result, molecules that are excluded at this stage according to the criteria detailed below do not require the far more expensive calculations associated with S_1_ geometry optimization. If the T_1_ geometry optimization does not converge within 200 cycles, the geometry corresponding to the lowest energy encountered during the optimization is identified, and its connectivity matrix is computed. If this matrix differs from that of the S_0_ geometry, the molecule is excluded. If connectivity is preserved, a second geometry optimization cycle is performed with different convergence criteria (see Section S3 of the SI for further details). Molecules for which geometry optimization fails to converge after this second optimization step are excluded from the workflow. Different connectivity matrices between electronic states do not represent a failure of the methodology; rather, they provide important information about the systems under investigation. In such cases, the molecule may undergo significant structural rearrangements upon excitation, such as decomposition or tautomerization.

A single-point TDDFT calculation for T_1_ at the equilibrium geometry obtained from ΔSCF optimization is performed. If a negative excitation energy is obtained or significant de-excitation amplitudes are detected, the molecule is flagged and excluded. It is worth emphasizing that geometry optimization is essential to reveal de-excitations or negative transition energies.

The more computationally demanding optimization of the S_1_ state, using TDDFT and starting from the equilibrium geometry of the T_1_ state, is performed next. A similar procedure to the one employed for the T_1_ state is applied to handle potential connectivity changes, ensure successful geometry optimization, and check for de-excitation contributions and anomalously low excitation energies also for S_1_. If the connectivity matrices between S_1_ and T_1_ states differ, the molecule is flagged and excluded from further analysis. Indeed, changes in molecular connectivity indicate that the S_1_ and T_1_ states no longer correspond to different electronic configurations of the same molecular framework. Under these conditions, the vibronic treatment required to evaluate the S_1_ → T_1_ Franck–Condon weighted density of states is no longer physically meaningful. Then, frequency calculations are performed for the remaining molecules in their S_1_ and T_1_ electronic states at the same level of theory used for the optimization process. If imaginary frequencies are detected, a single attempt is made to remove them by reoptimizing the molecule after displacement along the eigenvector corresponding to the largest imaginary frequency. Molecules that still exhibit imaginary frequencies are subsequently discarded.

At this stage, RISC rate constants can be computed from eqs 1 and 2. The required S_1_–T_1_ spin–orbit coupling (SOC) values were computed using ORCA software at the M06-2X/6-31G** level within the atomic mean-field integral (AMFI) approximation.^59^ In particular, SOC components were computed at the S_1_ equilibrium geometry using the Tamm–Dancoff approximation (TDA). The FCWDs, instead, were obtained using a development version of MolFC.^60^ Throughout this work, the internal representation of normal coordinates was adopted, as it mitigates spurious couplings in the FCWD arising from unphysical shifts in stretching coordinates that are induced by angular coordinate displacements during the geometry change associated with electronic transition.^61^

## Results

The automated protocol described above was tested for 70 candidate systems. Ground-state geometry optimizations and frequency calculations converged successfully for all of the molecules extracted from the DIADEM database. Furthermore, within this set of molecules, TDDFT vertical excitation calculations for the T_1_ and S_1_ states did not reveal any de-excitation contributions or anomalously low vertical excitation energies. Following the criteria described earlier, five compounds were excluded due to significant structural discrepancies between the S_0_ equilibrium geometry and the T_1_-state equilibrium geometry, as identified by comparing their connectivity matrices. In addition, 14 molecules were discarded because they exhibited de-excitations and/or a T_1_ excited-state minimum lying below the S_0_ state upon performing TDDFT single-point calculations for T_1_ at its equilibrium geometry.

The presence of highly flexible molecules in the data set is often associated with large-amplitude vibrational modes characterized by flat potential energy surfaces. Although the most rigorous approach would be to explicitly account for their anharmonic nature,^61^ a fully anharmonic treatment would be computationally prohibitive for systems of this size and therefore incompatible with an HTVS approach. Hence, we employed a mode-exclusion strategy to compute the RISC rate using an algorithm implemented and validated in our earlier work.^23^ This algorithm ensures consistency across the data set and can be applied automatically to multiple molecules without human intervention to ensure there is no hand-picking of the excluded modes. In particular, modes with wavenumbers below 100 cm^–1^ are excluded by default, and the algorithm automatically identifies whether additional modes should be discarded as well. Despite the introduction of this protocol, certain systems exhibited strongly anharmonic modes that precluded the achievement of reliable results, even after applying the exclusion procedure. Consequently, six additional molecules were discarded from the final analysis. A table listing the ZINC IDs of the excluded molecules, together with the corresponding reasons for exclusion, is provided in the Supporting Information (Table S1). Eventually, this procedure reduced the pool of candidates to 45 molecules, based on the criteria discussed above (Figure 2). A more in-depth investigation of the excluded systems is feasible but lies beyond the scope of this work, as it would require specialized methodologies tailored to the specific issues involved, such as isomerization, multiconfigurational effects, and anharmonicity.

**Figure 2:**
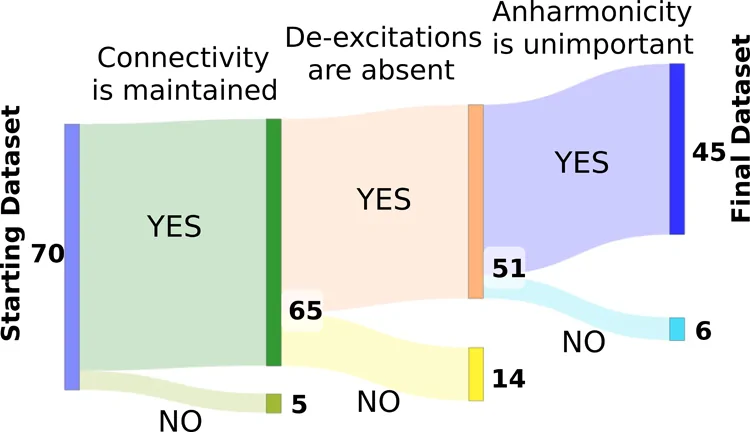
Diagram summarizing the automated screening process that led to 45 final candidates.

Figure 3 shows the RISC rates of the candidate molecules plotted as a function of Δ*E*_ST_, with the reorganization energy, λ, indicated by different colors and distinct spin–orbit coupling intervals represented by marker shapes. Here, λ values were calculated as the difference between the adiabatic Δ*E*_ST_ (S_1_–T_1_ at their own equilibrium geometry) and the vertical excitation energy associated with the S_1_ → T_1_ intersystem crossing transition, as obtained from TDDFT calculations. A full table of the results is presented in the Supporting Information (Table S2). As expected, the RISC process slows down as the singlet–triplet energy gap increases.^9–12,14–16^ This trend is accurately captured by our model, as evidenced by an *R* ^2^ = 0.9695 obtained from a linear fit of log_10_(*k*_RISC_) as a function of Δ*E*_ST_. From Figure 3, it is apparent that the most efficient molecules exhibit predicted adiabatic Δ*E*_ST_ values below 0.3 eV. Notably, although the initial screening was performed on molecules with vertical Δ*E*_ST_ < 0.5 eV, our calculations frequently yielded higher adiabatic values. These results indicate that, in future applications, the protocol could exclude molecules with adiabatic Δ*E*_ST_ exceeding 0.3 eV from further consideration.

**Figure 3:**
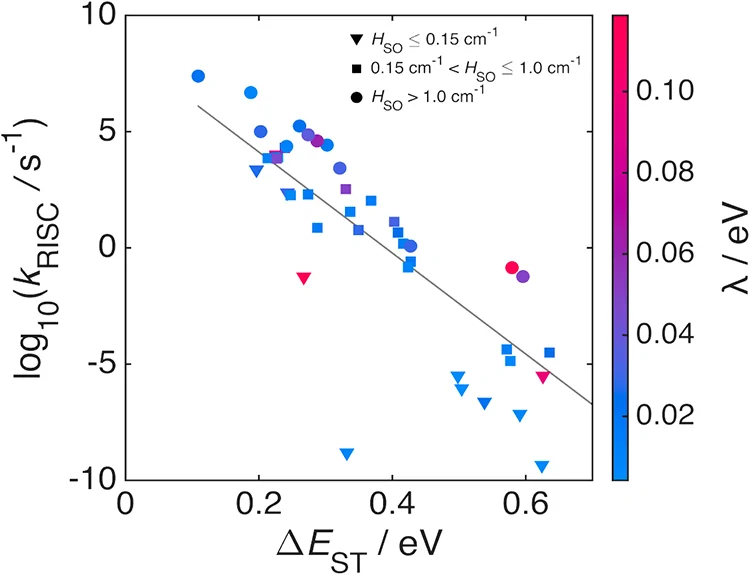
RISC rates (log_10_(*k*_RISC_)) of the candidate molecules as a function of the adiabatic singlet–triplet energy gap (Δ*E*_ST_). Colors indicate different values of the reorganization energy (λ), whereas marker shapes correspond to distinct intervals of spin–orbit coupling (*H*_SO_). The gray line shows a linear fit of log_10_(*k*_RISC_) versus Δ*E*_ST_.

All computed reorganization energies λ fall below 0.25 eV. In particular, while considering the most efficient systems, a value smaller than 0.05 eV appears to be a necessary, although not sufficient, condition for TADF efficiency. Since no frequency calculations are required, this quantity can be obtained at relatively low computational cost via a single additional vertical TDDFT calculation of T_1_ at the S_1_ equilibrium geometry. This may enable, in the future, the introduction of an additional filtering layer within the HTVS workflow, for instance, by discarding systems with reorganization energies larger than 0.10 eV.

No clear correlation between the spin–orbit coupling and the RISC rates emerges from these results, except for the observation that relatively high rates are only obtained for *H*_SO_ values larger than 1 cm^–1^. Overall, while a hierarchy exists among the parameters governing the RISC rate, with the singlet–triplet energy gap remaining the most influential, spin–orbit coupling and Franck–Condon terms can still modulate the rate over several orders of magnitude. However, Δ*E*_ST_ alone is not a reliable predictor of RISC efficiency, and explicit rate calculations provide essential information that cannot be inferred from the energy gap. For example, as reported in Table S2 of the Supporting Information, ZINC252620522 and ZINC16941977 exhibit relatively small Δ*E*_ST_ values of 0.23 and 0.27 eV, yet their RISC rates are extremely low, on the order of ∼10^–13^ and ∼10^–2^ s^–1^, respectively.

Among the 45 organic molecules considered, only four exhibit RISC rates exceeding 10^5^ s^–1^ and can thus be regarded as potential TADF emitters (Figure 4). Furthermore, a list of the indices of the modes excluded during the computation of the S_1_ → T_1_ FCWD for the last four molecules is given in Table S3 of the Supporting Information.

**Figure 4:**
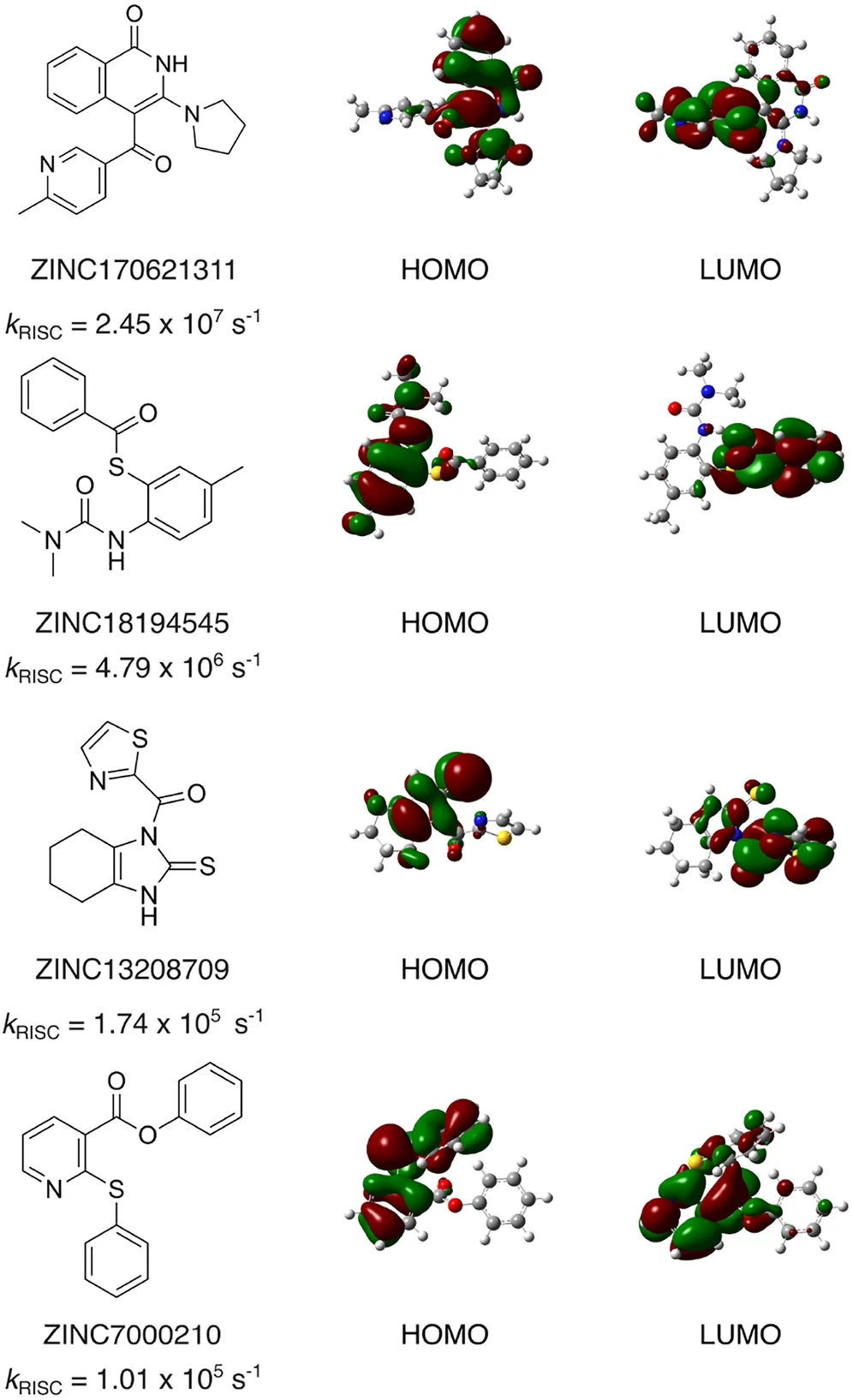
Left: Molecular structures, labeled with their ZINC IDs, displaying RISC rates higher than 10^5^ s^–1^. Right: isosurface contour plots of the HOMO and LUMO orbitals.

Notably, since the initial data set was not specifically designed for TADF applications, the selected molecules are, unsurprisingly, quite different from those typically considered in this field, which is a highly desirable feature for a screening procedure. Although applying the workflow to a TADF-oriented data set would likely increase the hit rate of TADF candidates, it would also reduce the chemical novelty of the results. Indeed, this chemical diversity makes the present data set a more informative and stringent test of the robustness of the workflow as an automated screening procedure. Inspection of their 2D structure does not reveal an obvious donor–acceptor character, such as the one normally imposed in other virtual screening studies,^62^ but analysis of the HOMO and LUMO orbitals involved in the lowest energy excitation reveals that three of the four compounds display a charge-transfer S1 and T1 excited state, normally associated with a small energy gap (Figure 4). An interesting feature of these four compounds lies in their excited-state equilibrium geometries. In all cases, the molecular structures can be described as composed of two fragments connected by a dihedral angle exceeding 60°, leading to nonplanar geometries with spatially separated conjugated units (see Figures S2–S5 in the Supporting Information). This is a feature found in the most common TADF molecules, such as 4CzIPN,^8^ and often justified by the requirement of creating charge-transfer excitations between orthogonal conjugated fragments.^63^ Concurrently, such nonplanar conjugated structures are known to promote larger spin–orbit couplings^64,65^ also in molecules with no heavy element, such as ZINC170621311. Furthermore, the latter features a core unit akin to benzophenone, a chromophore well-known to exhibit very fast ISC processes, particularly when the T_1_ state is involved.^66^ Molecule ZINC7000210 is the only one displaying a partial spatial overlap between HOMO and LUMO orbitals, but it is otherwise similar in structure. Finally, the relatively low reorganization energy associated with the RISC process is not clearly attributable to extreme delocalization (given the small size of the fragments) or to molecular rigidity (as conjugation is disrupted by structural distortions). Rather, it can be ascribed to the very similar bonding character of the S_1_ and T_1_ states, arising from similar single-electron configurations.

Fast RISC alone is not sufficient to ensure efficient TADF performance. Therefore, we also computed fluorescence FCWDs (Figures S6–S9 in the SI) and the corresponding radiative rates (Table S3 in the SI). The latter were obtained using the same methodology employed throughout this work, where the radiative emission rate, expressed in cgs Gaussis units, is given by^44^

8 $$k_{\mathrm{fluo}}\left(T\right)=\int_{0}^{\infty }\frac{4n^{2}\left|\left\langle S_{1}\right|\mathrm{\mu ̂}\right|S_{0}\rangle {|}^{2}}{3\hbar^{4}c^{3}}{\left(\hbar \omega \right)}^{3}F\left(E_{S_{0}}-E_{S_{1}}+\hbar \omega ,T\right)d\hbar \omega$$

where, consistent with the Strickler–Berg relation for radiative emission rates,^67^ *n* is the refractive index, ⟨*S*_1_|**μ̂**|*S*_0_⟩ is the electronic transition dipole moment for fluorescence, and (*E*_S_1__ – *E*_S_0__) is the purely electronic S_1_ ← S_0_ excitation energy. The resulting radiative rates further support our conclusions, as they are comparable to or larger than the corresponding RISC rates, thereby enabling efficient prompt and delayed fluorescence.

## Conclusions

We developed a fully automated screening algorithm that requires no manual intervention and was applied to a set of 70 organic molecules obtained after an initial screening from an original data set of 150,000 molecules, with the aim of identifying candidates that could act as efficient TADF emitters. The algorithm autonomously performs the initial elimination steps, excluding molecules from further analysis on the basis of (TD)­DFT-derived criteria such as excitation energies, the presence of de-excitations and so on. By ordering the calculations so that exclusion criteria of increasing computational cost are applied sequentially, the algorithm discards unsuitable candidates at the earliest possible stage, thereby steering the overall workflow toward the lowest achievable computational cost, while also providing an additional diagnostic advantage, as it identifies the specific issue responsible for the exclusion of each molecule. Such diagnostic capability allows one to distinguish between systems that can be reliably treated within the proposed framework and those that cannot, while also highlighting cases characterized by unconventional electronic structures. Although these latter systems may not be suitable for TADF applications, they may exhibit spectroscopic or dynamical properties of interest in other research contexts. In summary, the workflow presented in this work is capable of

- Automatically generating Gaussian input files and submitting the corresponding computations, both geometry optimization and frequency calculation;
- Handling errors across the workflow, raising controlled exceptions in response to infrastructure-level failures (e.g., failed job submissions);
- Handling typical issues arising in an optimization or frequency calculation by inspecting the output files and resubmitting the computations, if necessary, according to specific protocols;
- Parallelizing the output checking procedure;
- Flagging and discarding molecules from further computations according to specific criteria; and
- Computing the Franck–Condon weighted density of states and reverse intersystem crossing rates for the remaining candidates.

Following this screening procedure, a subset of 45 candidate molecules was retained. For these systems, our fully quantum-mechanical methodology based on Fermi’s golden rule was employed to compute RISC rates, thereby identifying four compounds with values consistent with efficient TADF behavior. The reliability of RISC rates as indicators of TADF efficiency was further supported by the analysis of equilibrium geometries and frontier orbitals, which revealed electronic characteristics consistent with TADF behavior for these molecules. Furthermore, our results indicate that selecting representative compounds from an unbiased data set and incorporating RISC rate information can provide valuable guidance for the design of new TADF molecules beyond the typically explored chemical space.

Beyond the identification of promising emitters, the analysis of this data set provided deeper insight into the key factors governing TADF efficiency. In this context, we were able not only to recover the expected relationship between the RISC rates and the adiabatic energy gap, but also to elucidate their dependence on the reorganization energy. Notably, our findings can be leveraged to further improve the material discovery process by introducing additional filtering steps based on the adiabatic Δ*E*_ST_ and the reorganization energy λ while relying on a fully automated algorithm explicitly designed with computational efficiency in mind.

## Supplementary Material



## Data Availability

The ground-state optimized structures, a sample computation log file, Duschinsky matrices for the last four candidates, and the Python scripts are available at https://github.com/teopizza/TADF_Pizza_Troisi_Capobianco.

## Notes

Molecular structures of all 70 molecules with corresponding ZINC IDs (Figure S1); optimized geometries of the S_1_ and T_1_ states for all molecules (Figures S2–S5); fluorescence FCWDs for the last four potentially efficient TADF molecules (Figures S6–S9); list of the 25 excluded molecules (Table S1); computed *k*_RISC_ values, adiabatic Δ*E*_ST_, reorganization energies, spin–orbit couplings, adiabatic *E*_S_1__ energies, and S_1_ ← S_0_ oscillator strengths for the 45 potential TADF candidates (Table S2); indices of the excluded modes in the computation of the S_1_ → T_1_ FCWD (Table S3); computed *k*_fluo_ values for the last four potentially efficient TADF molecules (Table S4); and description of the workflow used throughout this work (Section S3) (PDF)
