# Supplementary material for: Computation of Nonradiative Rates for High-Throughput Virtual Screening: Application to the Discovery of Potential TADF Molecules
Source: J Chem Theory Comput. 2026 Aug 20;22(17):9100–8. doi: 10.1021/acs.jctc.6c00881 (PMC13564352; doi:10.1021/acs.jctc.6c00881)
Supplement: Supplementary file 1 [file ct6c00881_si_001.pdf]

Supporting Information: Computation of Non-Radiative Rates for High-Throughput Virtual Screening: Application to Discovery of Potential TADF Molecules

Teodoro Pizza,[†] Alessandro Troisi,[*,‡] and Amedeo Capobianco[*,†]

†*Dipartimento di Chimica e Biologia "A. Zambelli", Università di Salerno, Via Giovanni Paolo II, 132, I-84084 Fisciano (SA), Italy*

‡*Department of Chemistry and Materials Innovation Factory, University of Liverpool, Liverpool, L69 7ZD, UK*

E-mail: a.troisi@liverpool.ac.uk; acapobianco@unisa.it

Contents

S1. Supplementary Figures **S-1**

S2. Supplementary Tables **S-11**

S3. Automated Workflow for $S_0/T_1/S_1$ Excited-State Characterization **S-14**

S1. Supplementary Figures

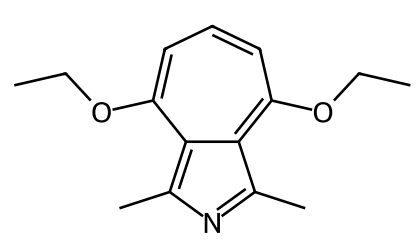

ZINC54457

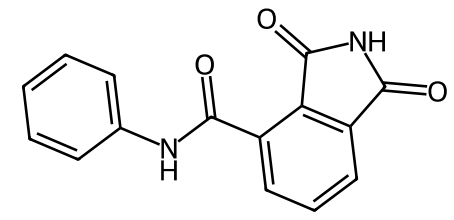

ZINC90720

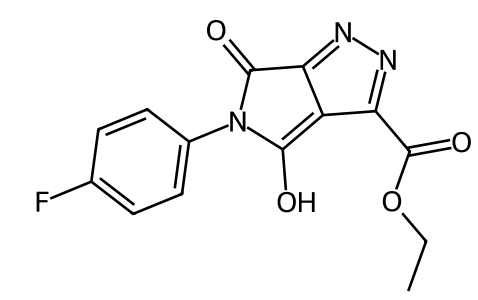

ZINC174513

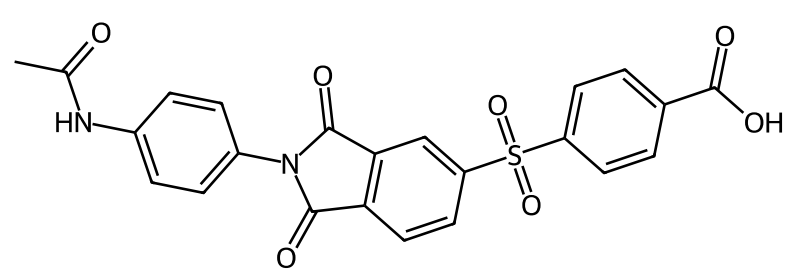

ZINC1210390

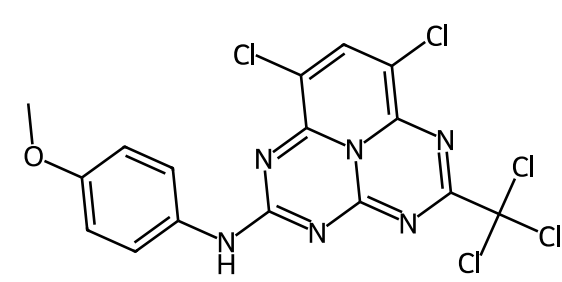

ZINC1586038

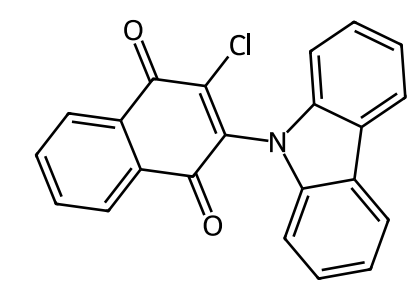

ZINC1672439

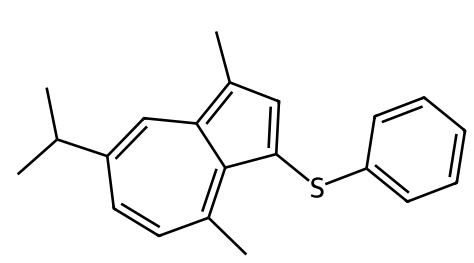

ZINC1686100

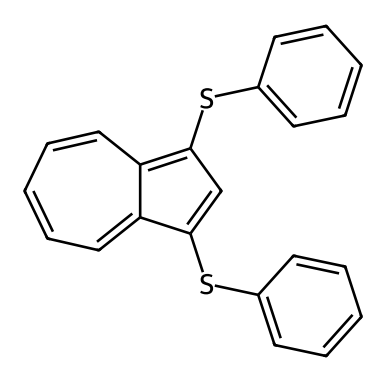

ZINC1686101

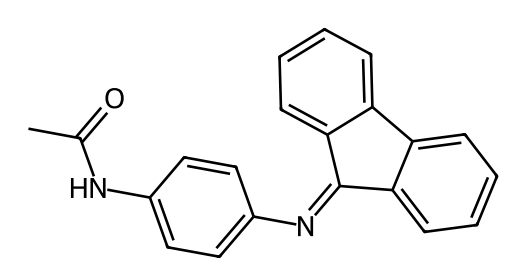

ZINC1724452

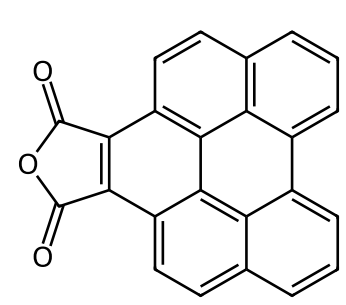

ZINC2154912

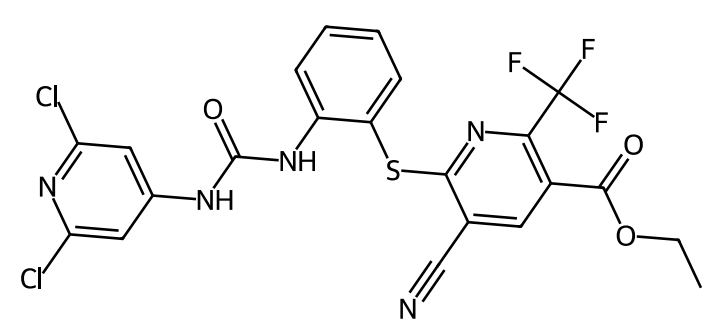

ZINC2559304

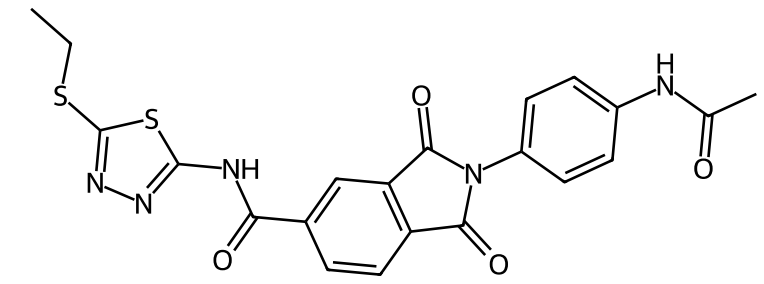

ZINC2654985

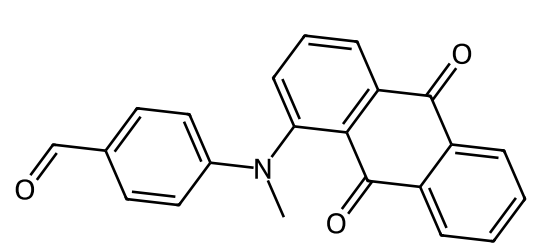

ZINC4088206

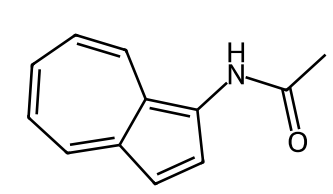

ZINC4947417

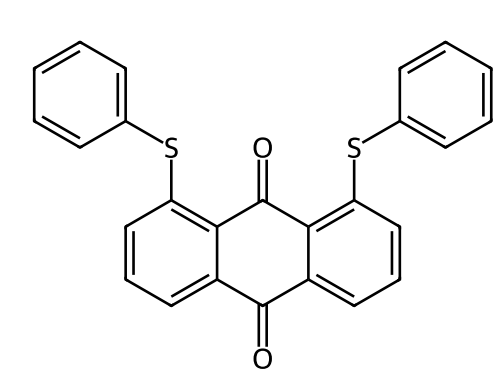

ZINC5124931

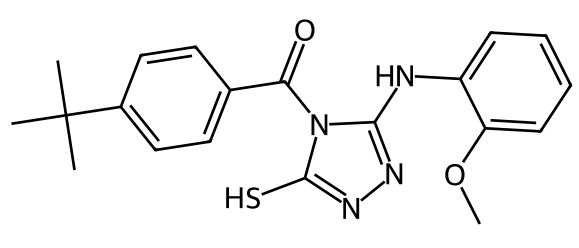

ZINC5460657

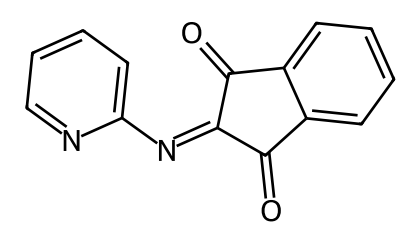

ZINC5800518

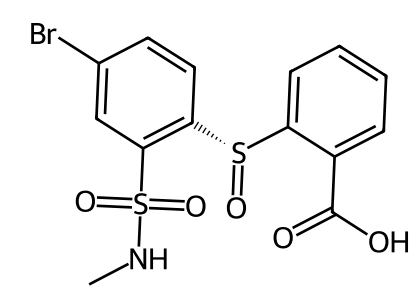

ZINC6161593

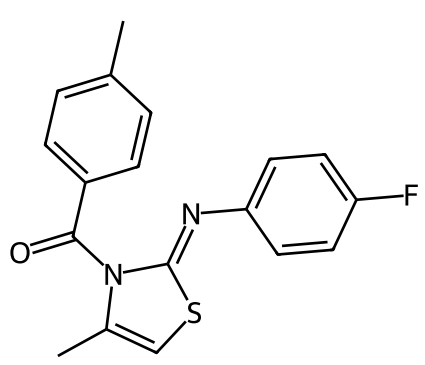

ZINC6204391

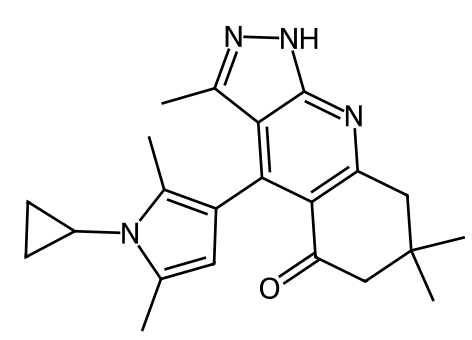

ZINC6995440

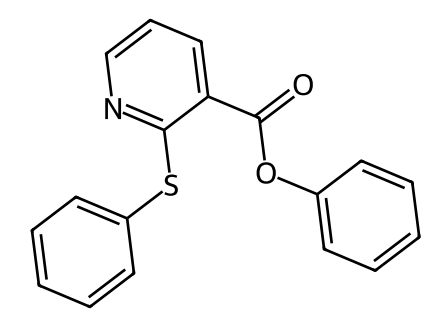

ZINC7000210

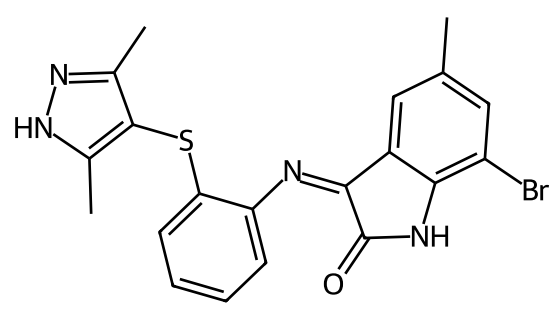

ZINC8386344

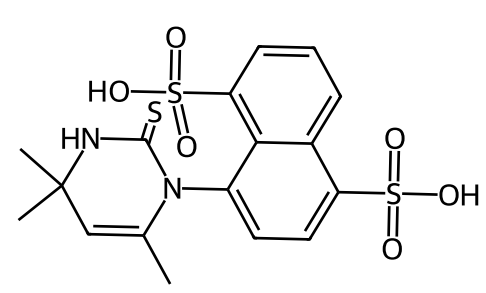

ZINC8602849

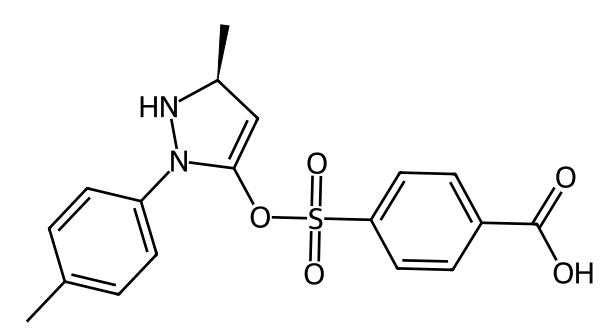

ZINC11691665

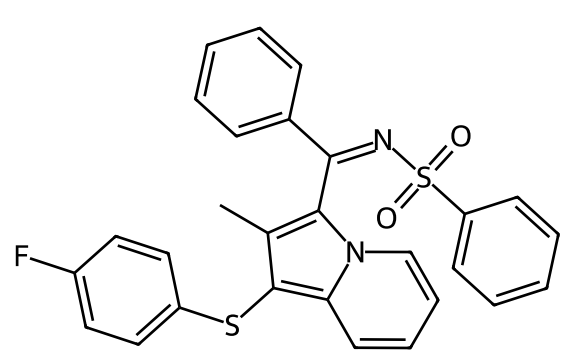

ZINC12558512

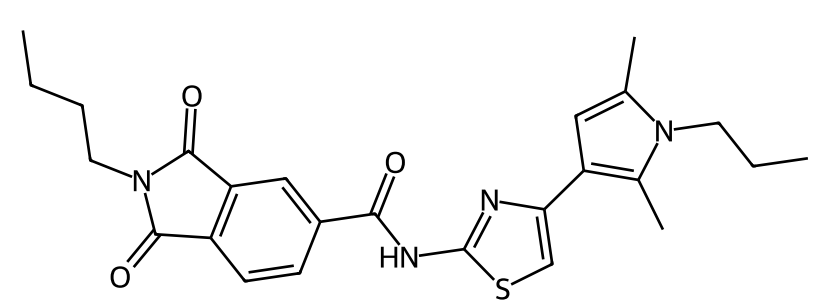

ZINC13149193

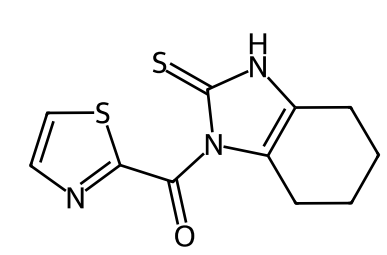

ZINC13208709

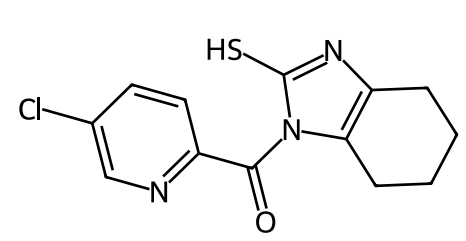

ZINC13208710

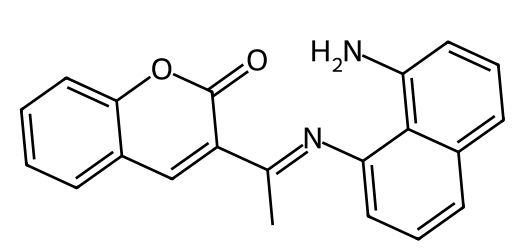

ZINC13407026

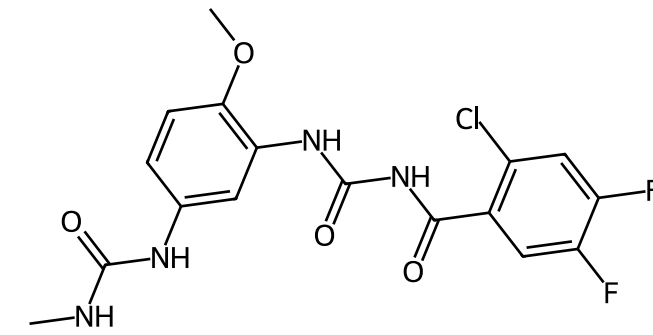

ZINC13673990

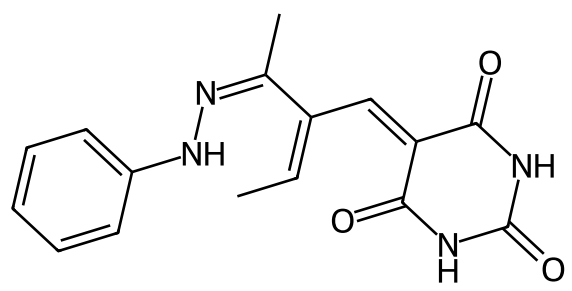

ZINC16137299

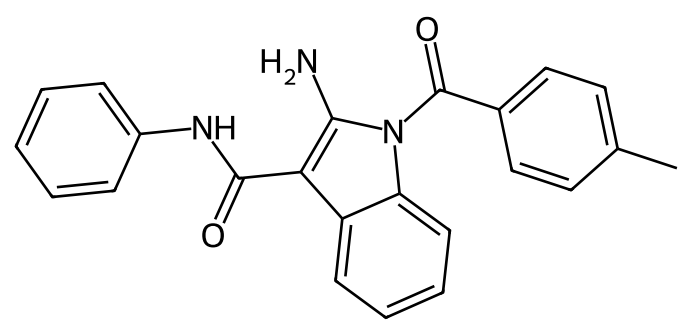

ZINC16778043

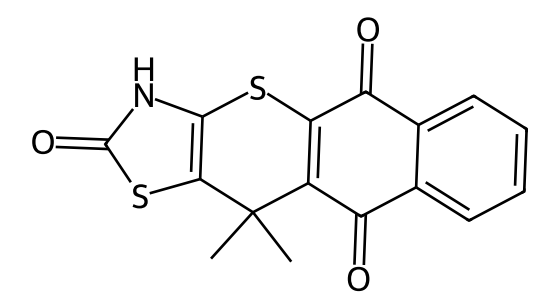

ZINC16779000

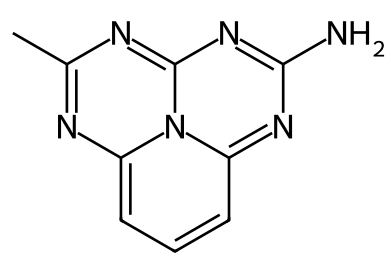

ZINC16941977

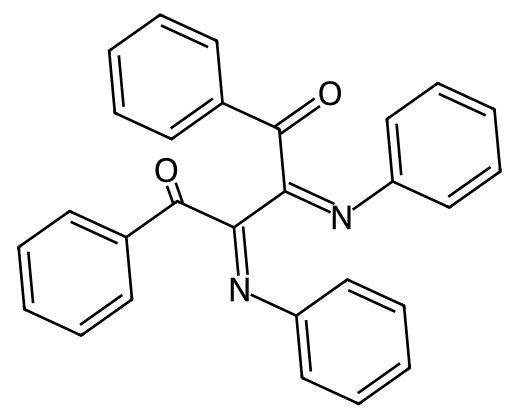

ZINC16954341

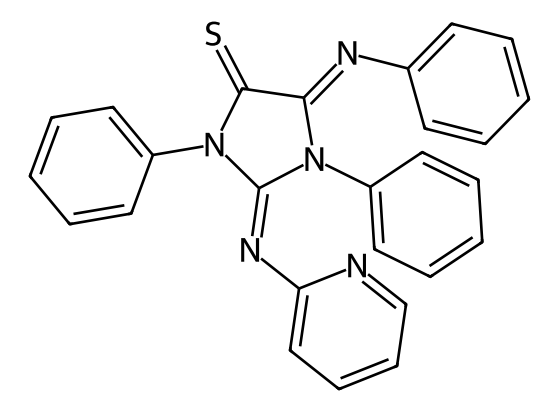

ZINC18156448

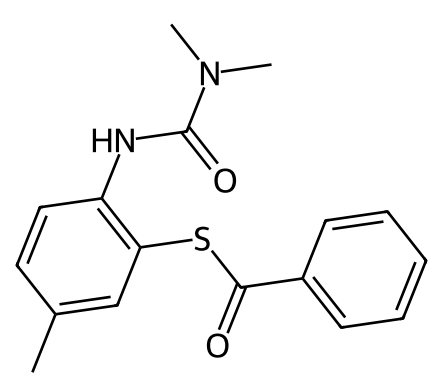

ZINC18194545

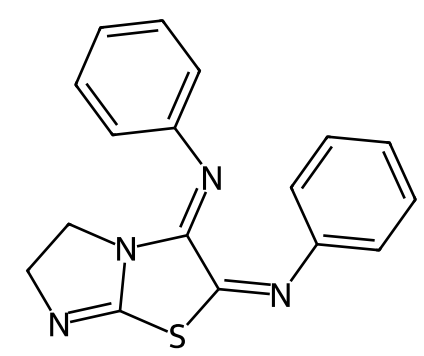

ZINC18252286

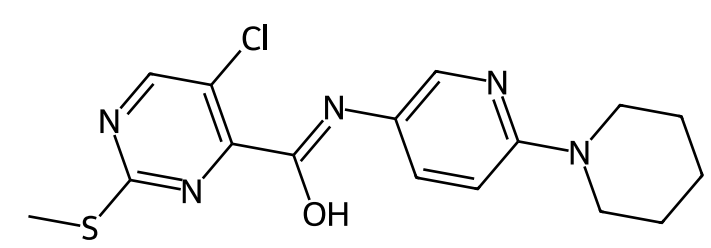

ZINC32753496

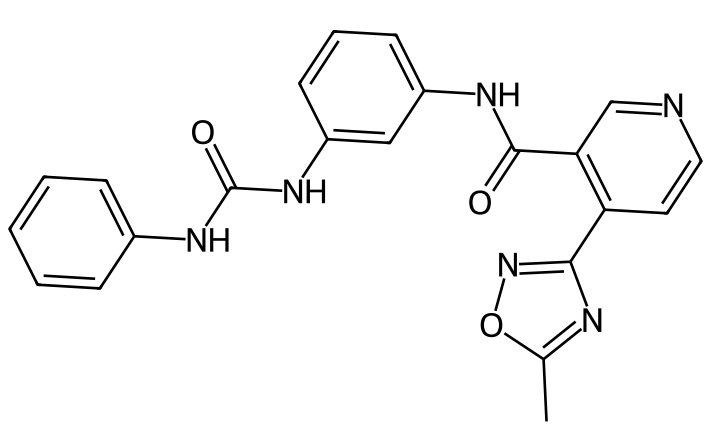

ZINC32916133

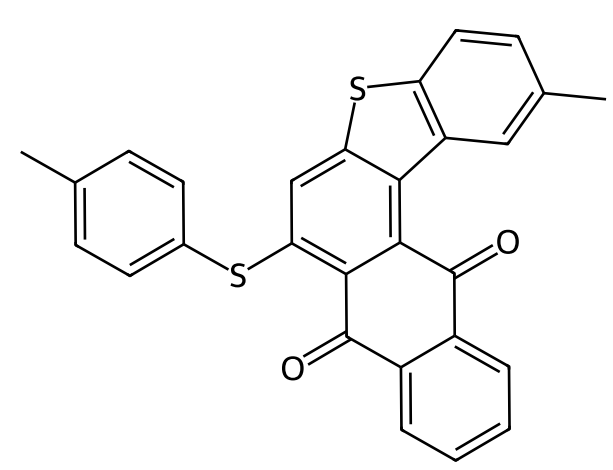

ZINC35323599

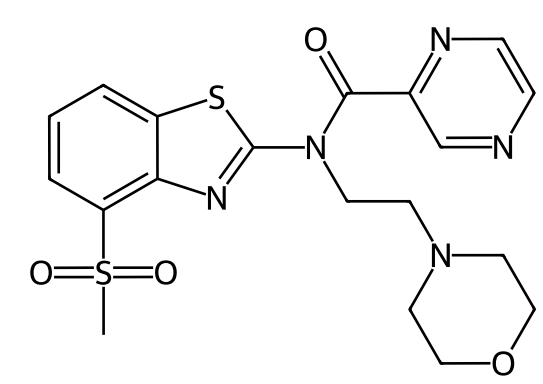

ZINC38845941

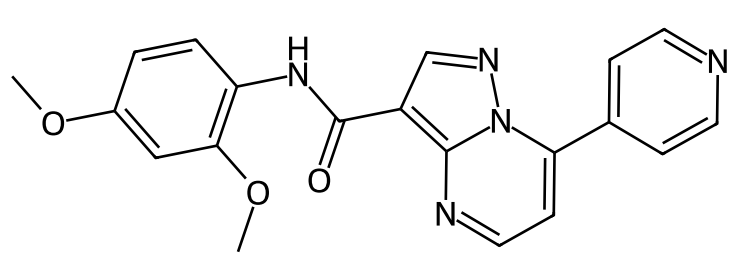

ZINC49002975

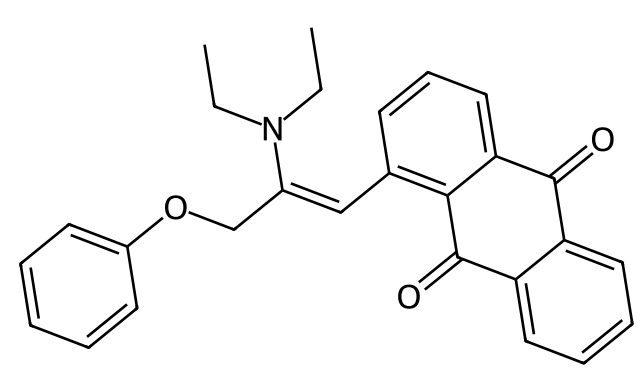

ZINC49717464

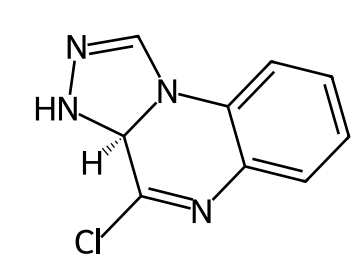

ZINC70503639

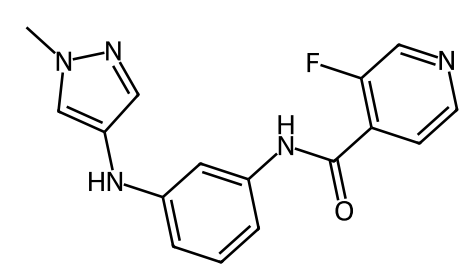

ZINC89756572

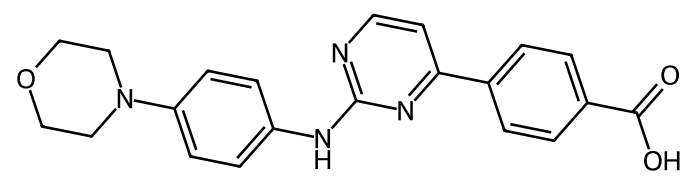

ZINC95080280

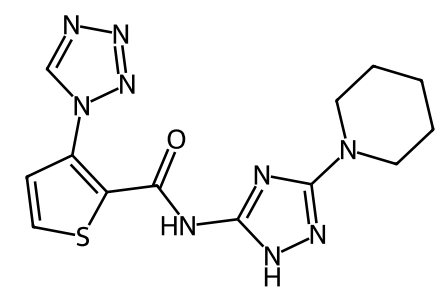

ZINC95394875

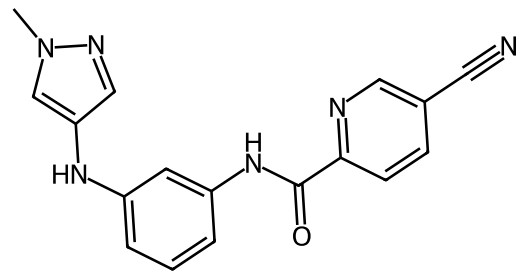

ZINC96449961

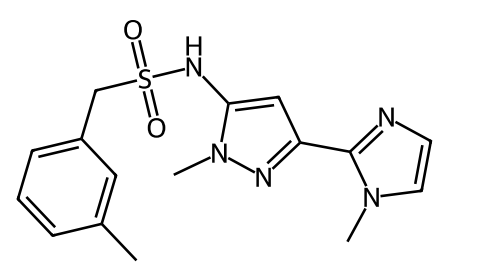

ZINC97140300

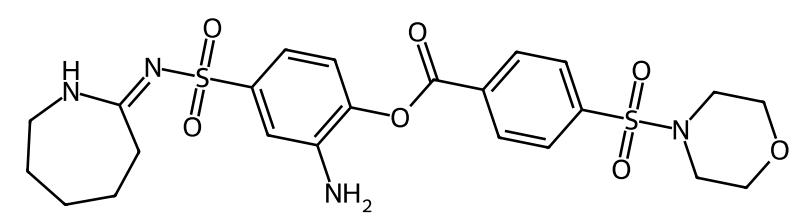

ZINC97981253

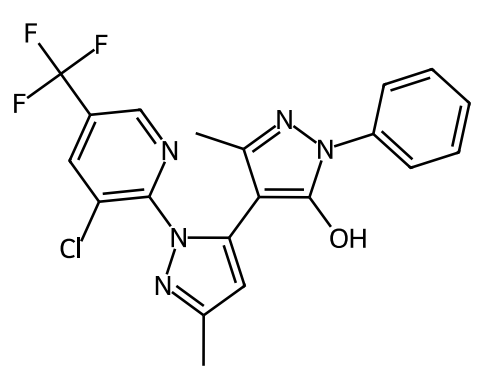

ZINC100920836

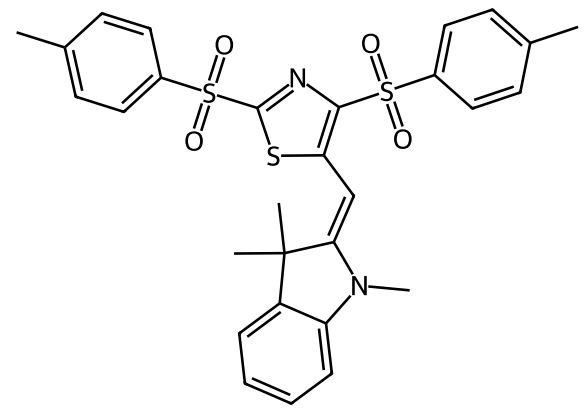

ZINC102934261

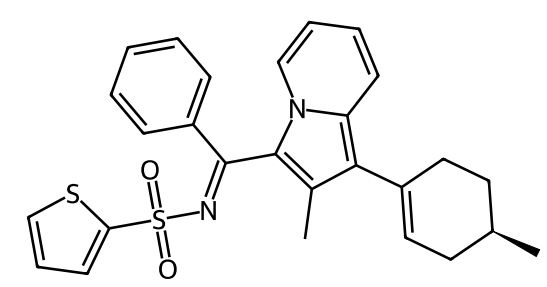

ZINC102941251

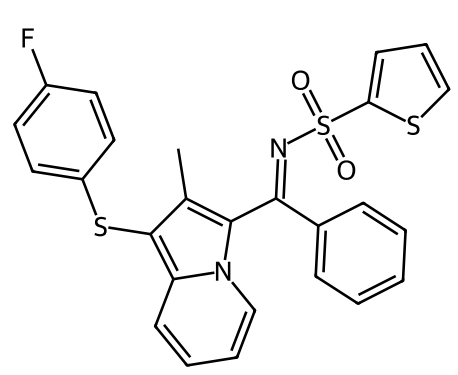

ZINC102941545

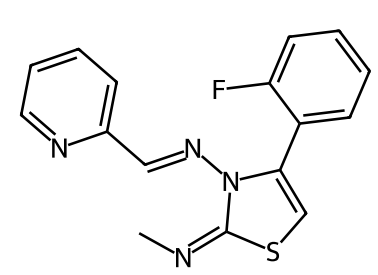

ZINC102960240

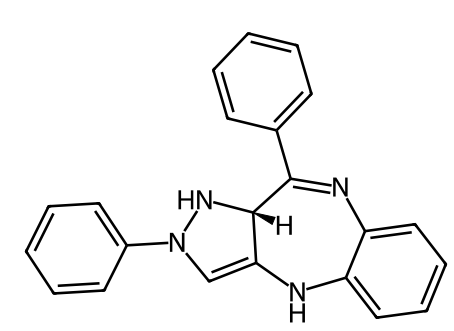

ZINC104646888

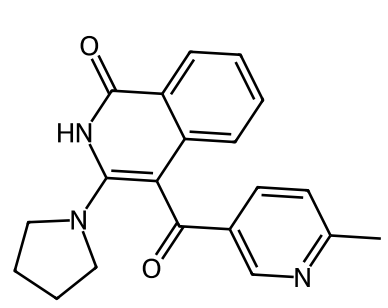

ZINC170621311

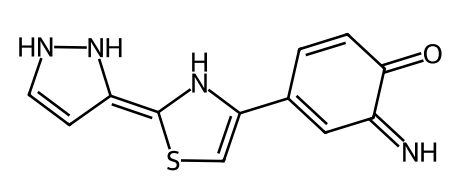

ZINC211601571

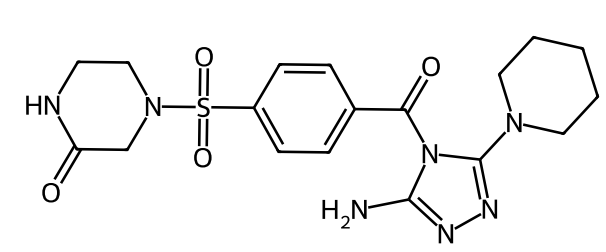

ZINC242389597

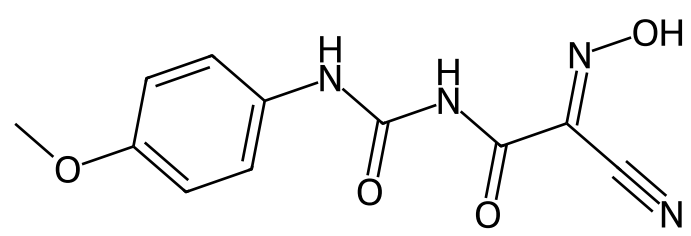

ZINC252620522

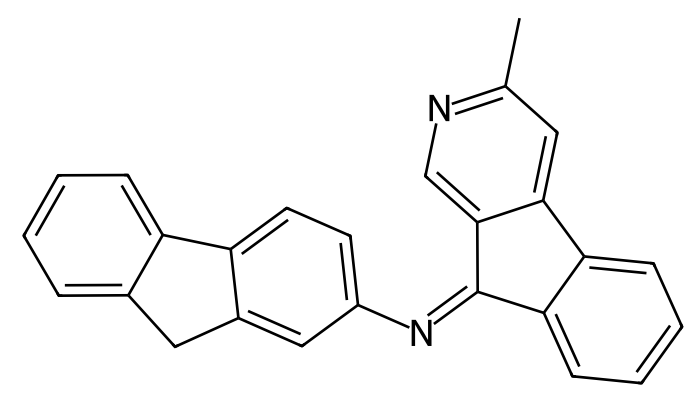

ZINC252630661

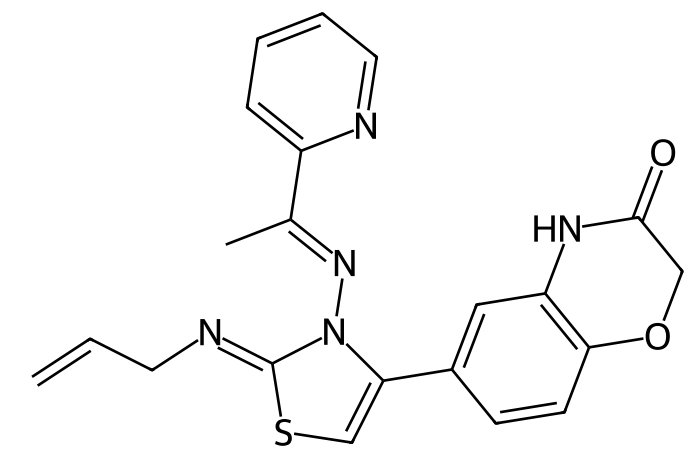

ZINC254808309

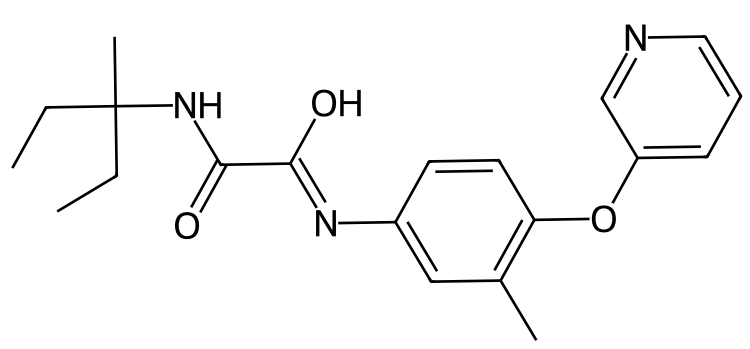

ZINC328596659

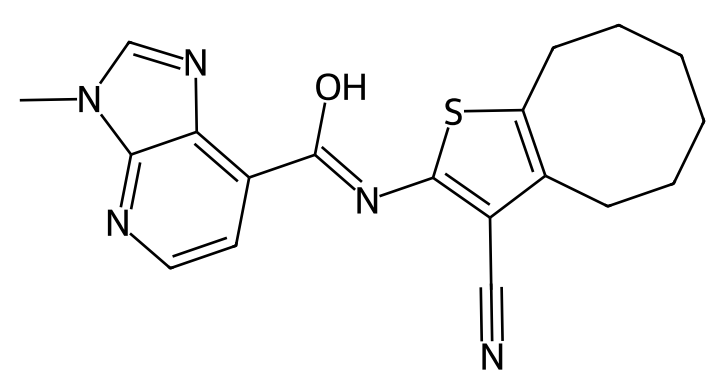

ZINC329325393

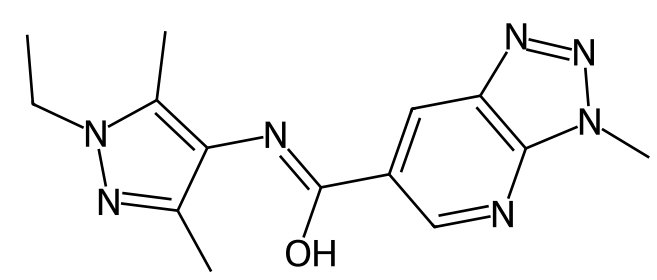

ZINC329655078

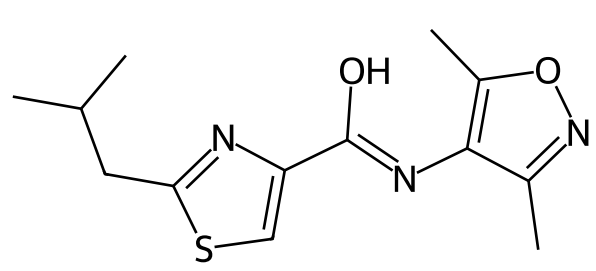

ZINC330494462

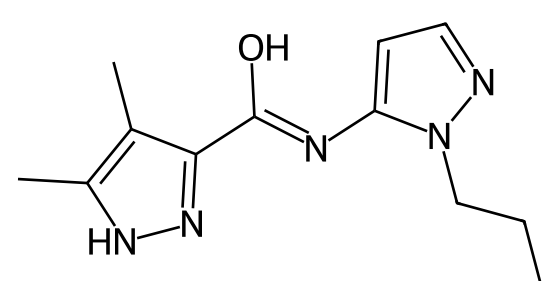

ZINC330824312

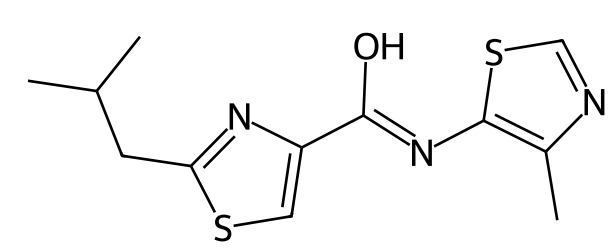

ZINC331025441

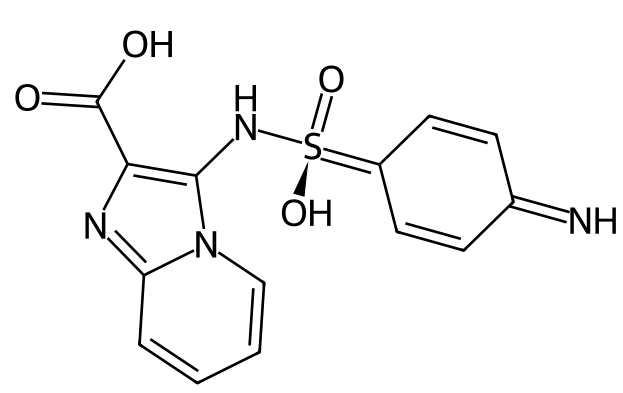

ZINC409415847

Figure S1: Molecular structures, together with their ZINC IDs , for all 70 candidates.

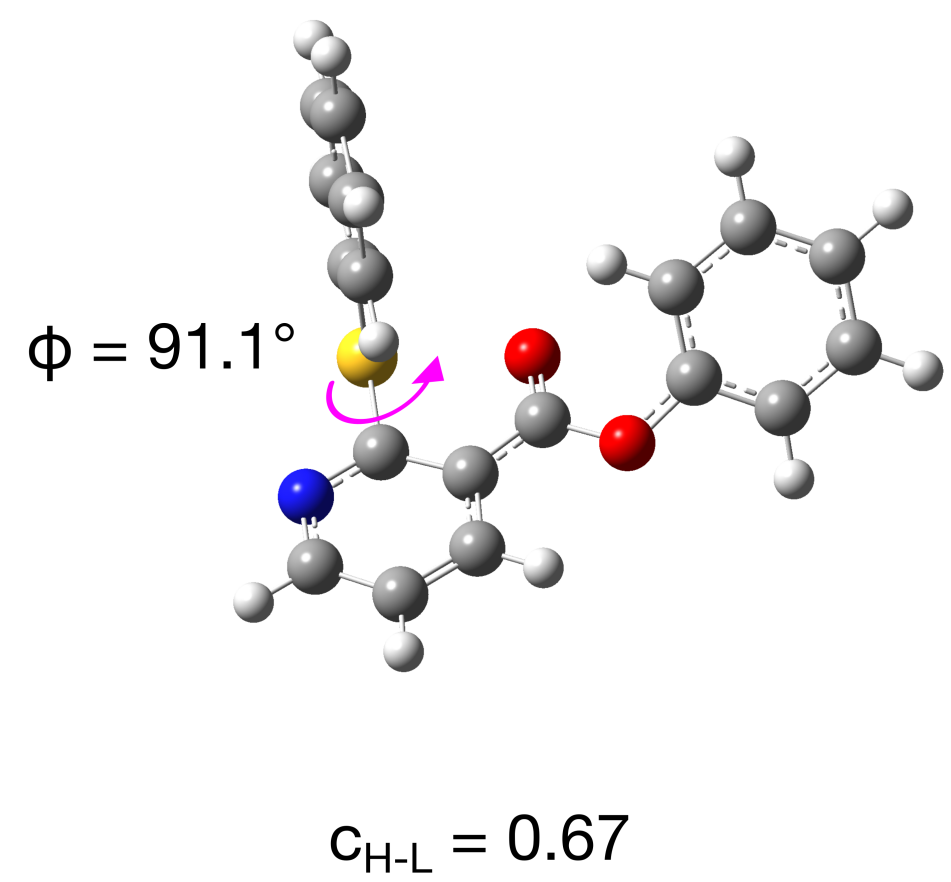

Figure S2: Optimized geometry of ZINC7000210 in its S_1 (left) and T_1 (right) excited states, together with the corresponding dihedral angles ϕ and HOMO–LUMO excitation amplitudes c_{H-L}.

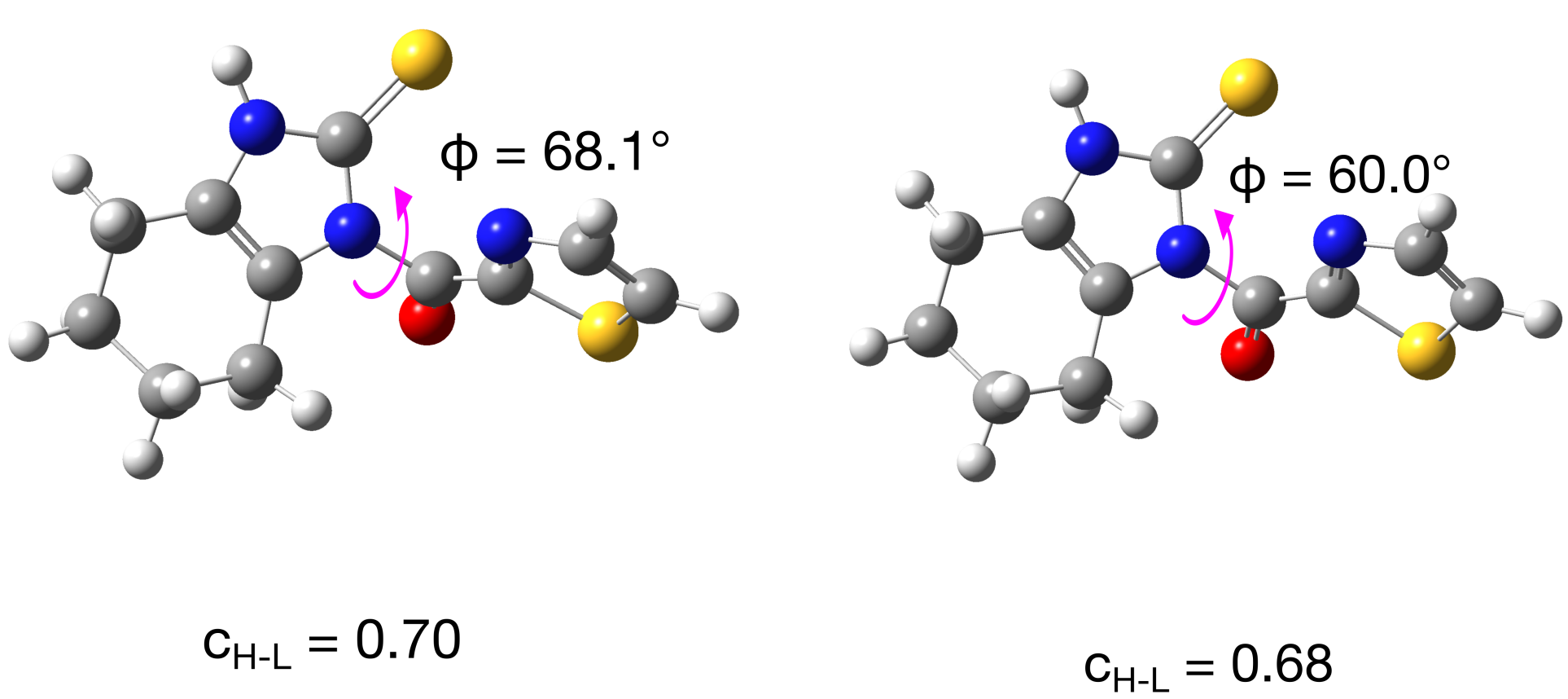

Figure S3: Optimized geometry of ZINC13208709 in its S_1 (left) and T_1 (right) excited states, together with the corresponding dihedral angles ϕ and HOMO–LUMO excitation amplitudes c_{H-L}.

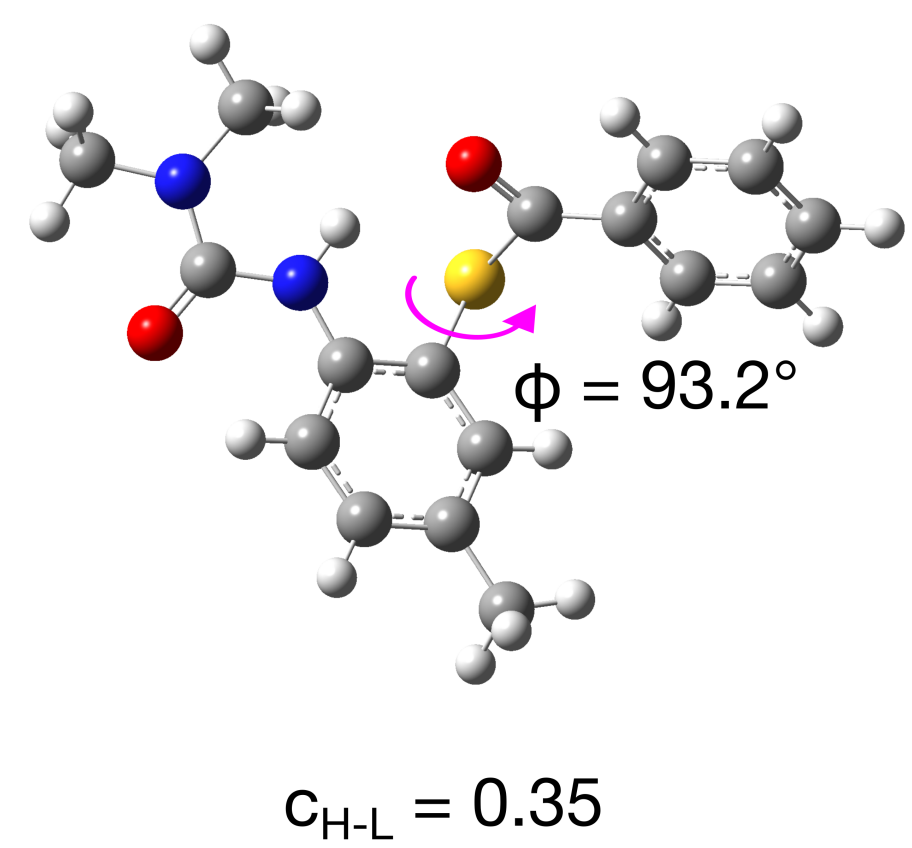

Figure S4: Optimized geometry of ZINC18194545 in its S_1 (left) and T_1 (right) excited states, together with the corresponding dihedral angles ϕ and HOMO–LUMO excitation amplitudes c_{H-L}.

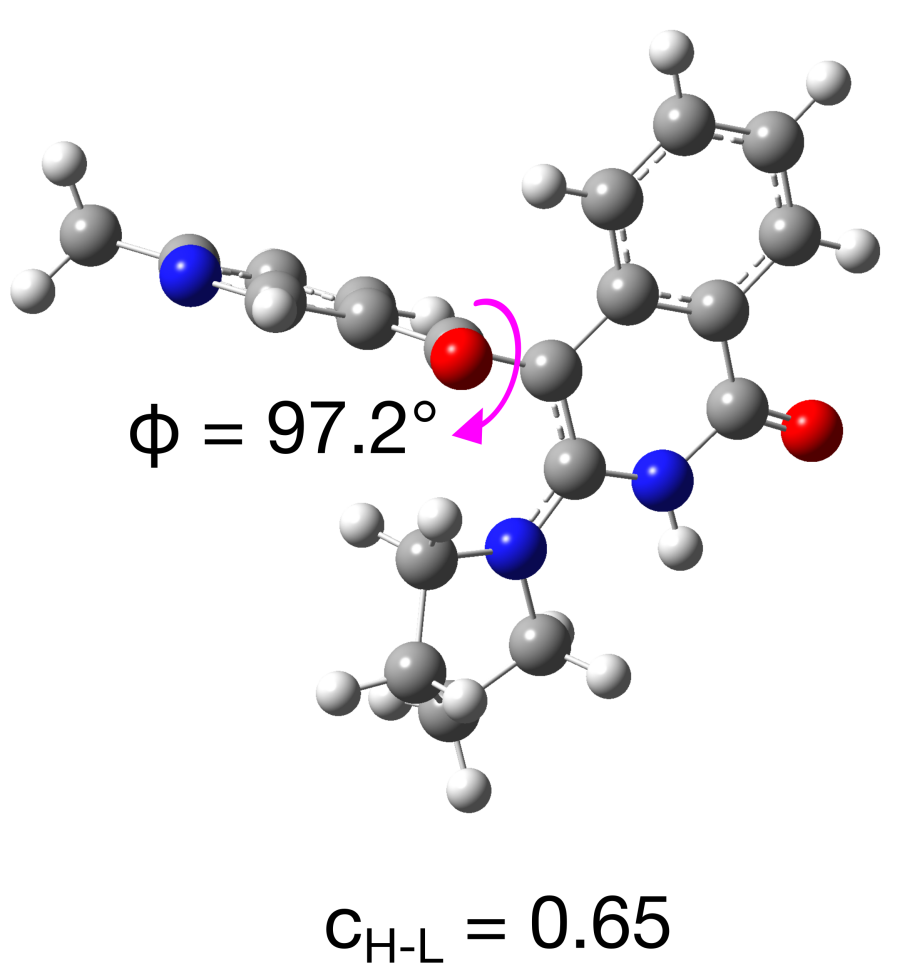

Figure S5: Optimized geometry of ZINC170621311 in its S_1 (left) and T_1 (right) excited states, together with the corresponding dihedral angles ϕ and HOMO–LUMO excitation amplitudes c_{H-L}.

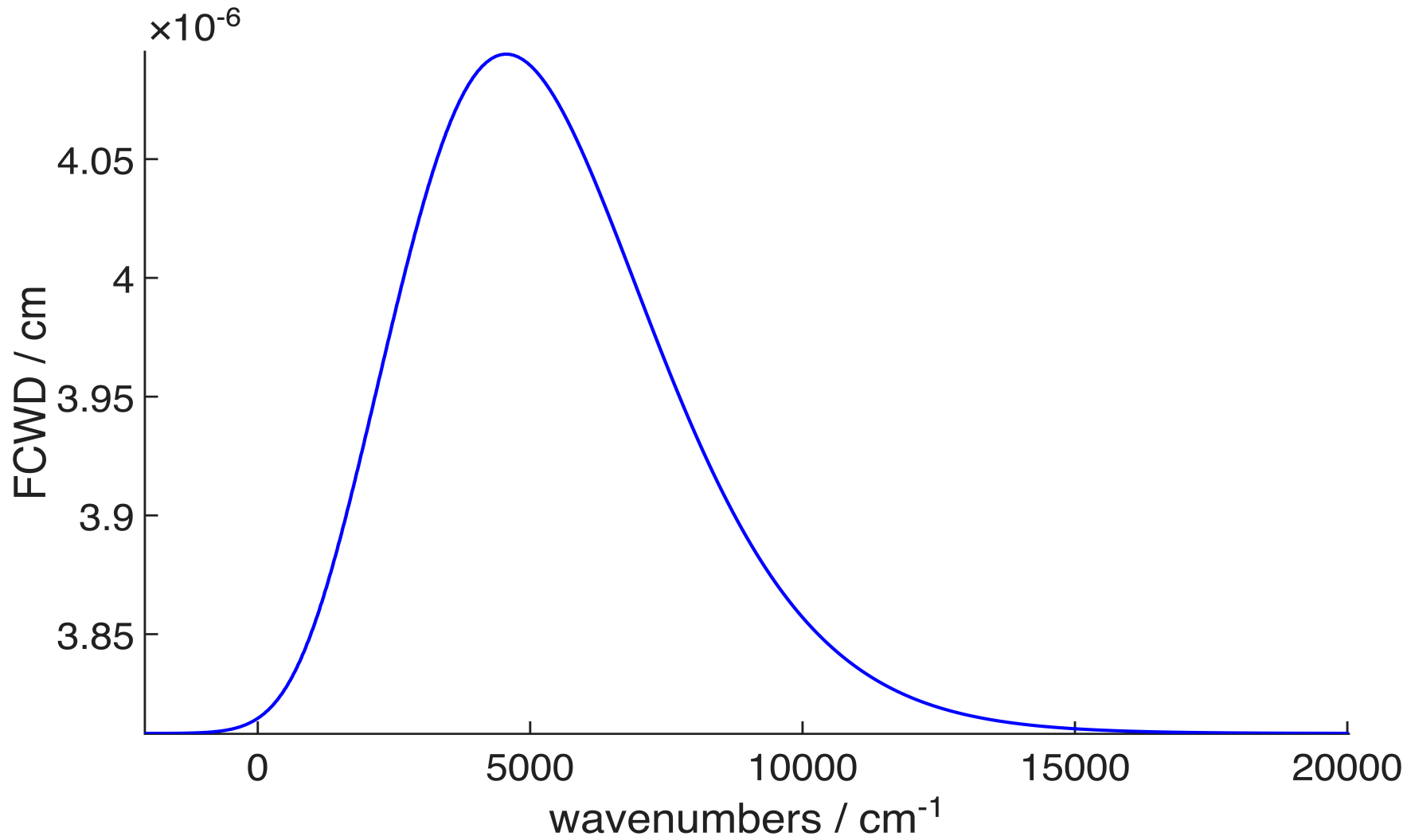

Figure S6: Predicted ZINC7000210 fluorescence FCWD.

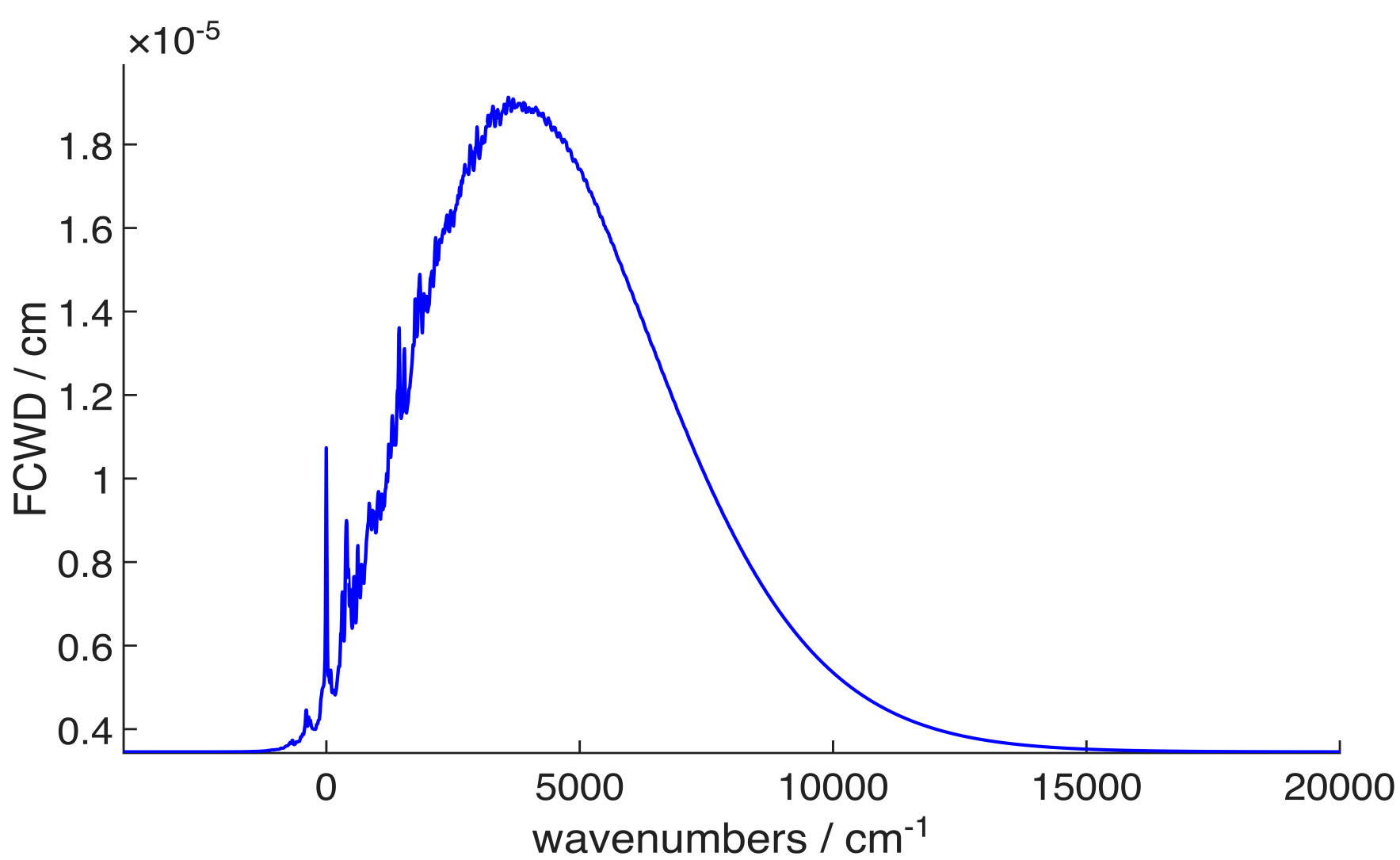

Figure S7: Predicted ZINC13208709 fluorescence FCWD.

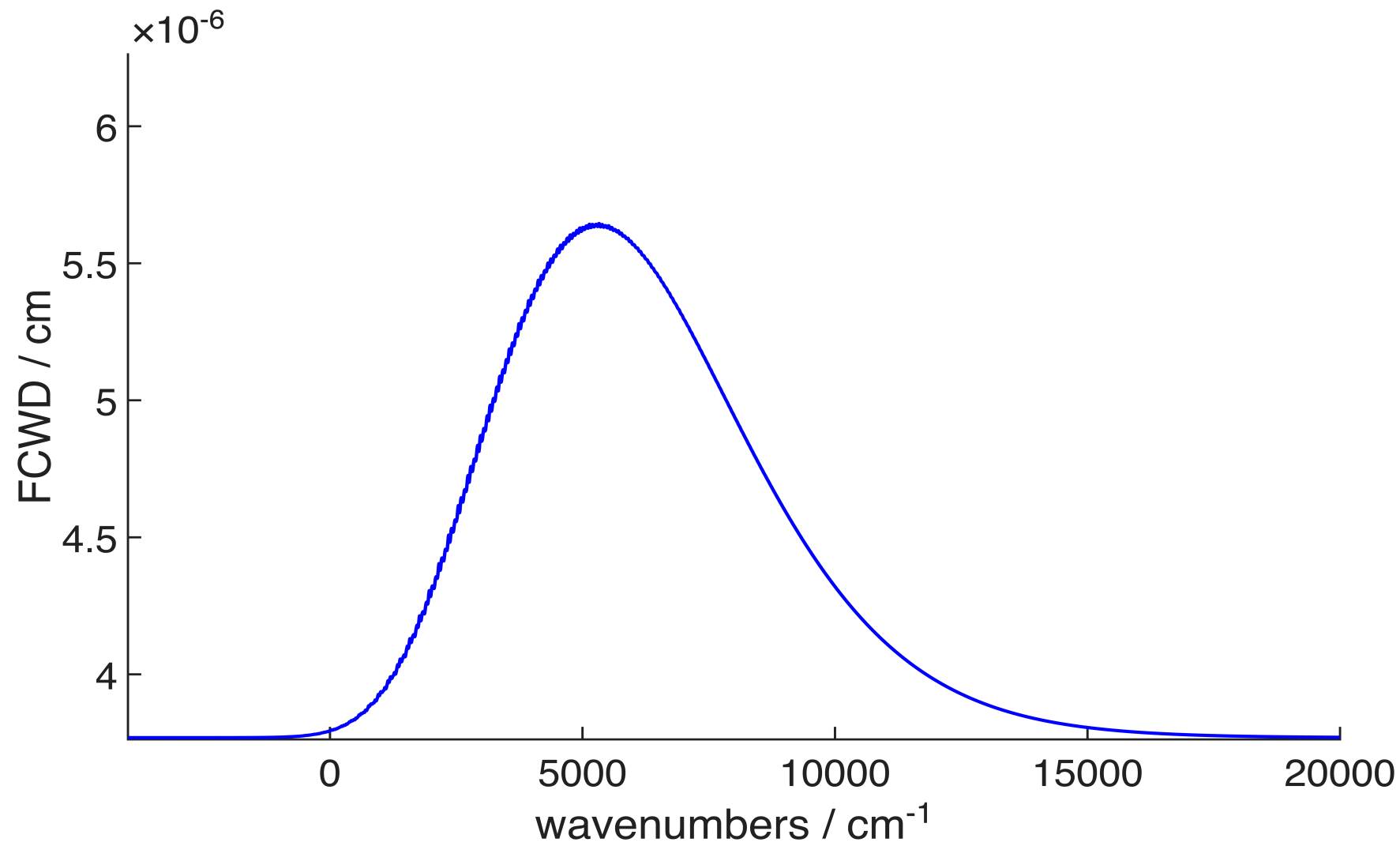

Figure S8: Predicted ZINC18194545 fluorescence FCWD.

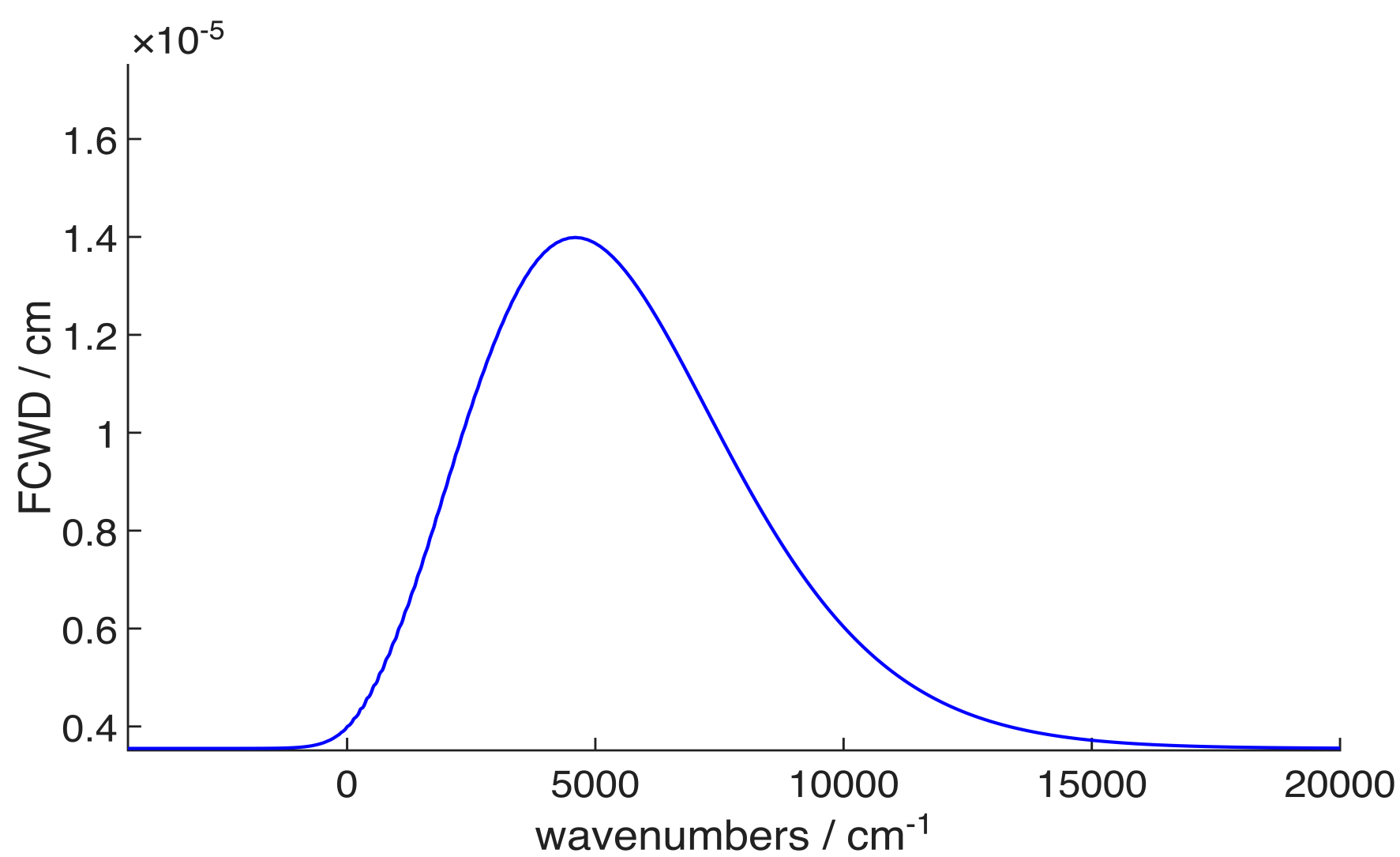

Figure S9: Predicted ZINC170621311 fluorescence FCWD.

S2. Supplementary Tables

Table S1: List of the 25 excluded molecules with the reason for their exclusion from the screening process.

ZINC ID	Reason for exclusion
ZINC6995440	Molecule removed due to differences in connectivity between S_0 and T_1
ZINC13673990	Molecule removed due to differences in connectivity between S_0 and T_1
ZINC100920836	Molecule removed due to differences in connectivity between S_0 and T_1
ZINC409415847	Molecule removed due to differences in connectivity between S_0 and T_1
ZINC90720	Molecule removed due to differences in connectivity between S_0 and T_1
ZINC1724452	Molecule removed due to de-excitations
ZINC5800518	Molecule removed due to de-excitations
ZINC8386344	Molecule removed due to de-excitations
ZINC11691665	Molecule removed due to de-excitations
ZINC13407026	Molecule removed due to de-excitations
ZINC16954341	Molecule removed due to de-excitations
ZINC18156448	Molecule removed due to de-excitations
ZINC32753496	Molecule removed due to de-excitations
ZINC102960240	Molecule removed due to de-excitations
ZINC211601571	Molecule removed due to de-excitations
ZINC252630661	Molecule removed due to de-excitations
ZINC254808309	Molecule removed due to de-excitations
ZINC18252286	Molecule removed due to de-excitations
ZINC329655078	Molecule removed due to de-excitations
ZINC1586038	Molecule removed due to the presence of strongly anharmonic modes
ZINC1672439	Molecule removed due to the presence of strongly anharmonic modes
ZINC2559304	Molecule removed due to the presence of strongly anharmonic modes
ZINC16137299	Molecule removed due to the presence of strongly anharmonic modes
ZINC97981253	Molecule removed due to the presence of strongly anharmonic modes
ZINC102934261	Molecule removed due to the presence of strongly anharmonic modes

Table S2: Computed k_{RISC} (s^{-1}) , adiabatic ΔE_{ST} (eV), λ (eV), H_{SO} (cm^{-1}), adiabatic $S_1 \leftarrow S_0$ excitation energies (E_{S_1}, eV) and the $S_1 \leftarrow S_0$ oscillator strength (f_{OSC}) for the 45 potential TADF candidates identified in this work.

ZINC ID	k_{RISC}	ΔE_{ST}	λ	H_{SO} (cm^{-1})	E_{S_1}	f_{OSC}
ZINC54457	1.55×10^{-9}	0.33	8.1×10^{-3}	6.69×10^{-6}	2.02	0.0111
ZINC174513	2.36×10^{2}	0.24	3.04×10^{-2}	0.13	2.80	0.0620
ZINC1210390	8.94×10^{-7}	0.50	4.2×10^{-3}	5.0×10^{-2}	2.95	0.0111
ZINC1686100	2.01×10^{2}	0.27	1.51×10^{-2}	0.29	1.96	0.0078
ZINC1686101	1.84×10^{2}	0.25	5.4×10^{-3}	0.43	1.90	0.0034
ZINC2154912	3.09×10^{-6}	0.63	9.76×10^{-2}	4.0×10^{-2}	3.03	0.2599
ZINC2654985	3.11×10^{-6}	0.50	4.6×10^{-3}	5.74×10^{-2}	2.99	0.0134
ZINC4088206	5.85×10^{0}	0.35	2.88×10^{-2}	0.29	2.40	0.0056
ZINC4947417	7.31	0.29	1.64×10^{-2}	0.17	1.93	0.0061
ZINC5124931	3.59×10^{1}	0.34	1.40×10^{-2}	0.96	2.33	0.0002
ZINC5460657	4.57×10^{0}	0.41	2.54×10^{-2}	0.84	3.08	0.0510
ZINC6161593	1.19×10^{0}	0.43	2.96×10^{-2}	1.92	3.62	0.0223
ZINC7000210	1.01×10^{5}	0.20	2.84×10^{-2}	1.45	3.33	0.0004
ZINC8602849	5.86×10^{-2}	0.60	4.98×10^{-2}	8.98	3.12	0.0441
ZINC13149193	3.15×10^{-5}	0.64	2.53×10^{-2}	0.44	3.07	0.0930
ZINC13208709	1.74×10^{5}	0.26	2.27×10^{-2}	4.80	2.46	0.0061
ZINC13208710	3.38×10^{2}	0.33	5.09×10^{-2}	0.68	2.75	0.0028
ZINC16778043	1.53×10^{0}	0.42	1.42×10^{-2}	0.42	3.17	0.0031
ZINC16779000	2.36×10^{-7}	0.54	2.20×10^{-2}	4.0×10^{-2}	2.21	0.0290
ZINC18194545	4.79×10^{6}	0.18	8.7×10^{-3}	6.64	3.36	0.0025
ZINC35323599	1.07×10^{2}	0.37	1.39×10^{-2}	0.80	2.21	0.0001
ZINC38845941	9.23×10^{3}	0.22	8.68×10^{-2}	0.64	3.37	0.0029
ZINC49002975	4.48×10^{-10}	0.62	5.2×10^{-3}	1.41×10^{-2}	2.98	0.0400

Continued from previous page.

ZINC ID	k_{RISC}	ΔE_{ST}	λ	H_{SO}	E_{S_1}	f_{OSC}
ZINC49717464	1.32×10^{1}	0.40	3.19×10^{-2}	0.59	2.03	0.0042
ZINC70503639	2.68×10^{3}	0.32	3.21×10^{-2}	1.77	2.85	0.0015
ZINC89756572	4.29×10^{-5}	0.57	1.25×10^{-2}	0.76	3.37	0.0201
ZINC95080280	7.08×10^{-8}	0.59	7.3×10^{-3}	3.32×10^{-2}	2.97	0.0130
ZINC95394875	2.65×10^{-6}	0.77	0.25	0.95	3.17	0.0148
ZINC96449961	1.36×10^{-5}	0.58	6.1×10^{-3}	0.46	3.03	0.0186
ZINC97140300	3.30×10^{-17}	0.77	5.36×10^{-2}	0.22	4.79	0.6507
ZINC102941251	2.28×10^{3}	0.57	2.69×10^{-2}	0.12	2.14	0.0002
ZINC104646888	2.56×10^{-1}	0.59	1.84×10^{-2}	0.77	2.28	0.0258
ZINC170621311	2.45×10^{7}	0.24	2.09×10^{-2}	2.01	2.59	0.0021
ZINC242389597	1.45×10^{-1}	0.30	9.7×10^{-3}	0.27	2.29	0.0019
ZINC252620522	2.35×10^{-13}	0.23	3.76×10^{-2}	4.18×10^{-3}	3.03	0.1745
ZINC328596659	2.33×10^{4}	0.62	1.11×10^{-2}	2.19	2.58	0.0000
ZINC329325393	2.64×10^{4}	0.23	1.57×10^{-2}	3.53	2.04	0.0000
ZINC330494462	7.82×10^{3}	0.30	8.6×10^{-3}	1.00	2.53	0.0001
ZINC330824312	3.99×10^{4}	0.23	6.09×10^{-2}	3.76	2.73	0.0002
ZINC331025441	7.25×10^{3}	0.29	1.17×10^{-2}	0.75	2.41	0.0000
ZINC6204391	7.32×10^{4}	0.24	3.8×10^{-2}	3.15	2.87	0.0113
ZINC12558512	1.99×10^{4}	0.23	6.5×10^{-2}	0.86	2.24	0.0013
ZINC32916133	1.41×10^{-1}	0.58	0.12	5.86	4.16	0.0033
ZINC102941545	6.88×10^{3}	0.43	4.7×10^{-2}	0.43	2.28	0.0005
ZINC16941977	5.66×10^{-2}	0.27	0.12	1.0×10^{-2}	2.83	0.0290

Table S3: Indices of the excluded modes in the computation of the $S_1 \rightarrow T_1$ FCWD for the four most promising molecules, arranged in order of ascending wavenumber.

ZINC ID	Excluded modes indices
ZINC7000210	From 1 to 6 ($<$100 cm^{-1}) and 9, 10, 12, 13, 14
ZINC13208709	From 1 to 6 ($<$100 cm^{-1}) and 10
ZINC18194545	From 1 to 9 ($<$100 cm^{-1}) and 10
ZINC170621311	From 1 to 6 ($<$100 cm^{-1}); from 11 to 13, 15 to 19 and 26

Table S4: Computed fluorescence rate constants, k_{fluo} (s^{-1}), for the four more promising molecules.

ZINC ID	k_{fluo}
ZINC7000210	2.65×10^{7}
ZINC13208709	9.91×10^{6}
ZINC18194545	4.68×10^{6}
ZINC170621311	1.08×10^{7}

S3. Automated Workflow for $S_0/T_1/S_1$ Excited-State Characterization

- All geometry optimizations with redundant internal coordinates adopt `maxstep <= 10` (the Gaussian default is `maxstep=30`).
- Molecules can be flagged for further review based on specific criteria. A log file reporting the molecule identifier code and any applicable flags is generated automatically.
- Once a molecule has been identified as discarded, it is flagged and excluded from all subsequent steps.
- All steps are automated by Python scripts that parse Gaussian outputs, check connectivity, de-excitations, convergence, and frequencies, generate new inputs, collect molecule identifiers, and write reports. No human intervention is required.
- Delta SCF (DSCF) refers to the optimization of the triplet excited-state (T_1) geometry by performing a self-consistent-field calculation with an electron occupation targeting the T_1 state.

Workflow Summary

1. **Optimization of the S_0 geometry and frequency calculation**
   - Start from the initial input geometry.
   - If the optimization `opt(maxstep=10,maxcycle=200)` fails to converge due to PCM-related issues, apply the PCM Error Protocol. Otherwise, apply the following restart procedure (always starting from the geometry with the lowest energy found so far):
     a. `opt(maxstep=5,maxcycle=100,calcfc)`
     b. if that fails → `opt(cartesian,maxcycle=200)`

   c. if that fails → flag and discard the molecule.

- Compute the frequencies: if imaginary frequencies are detected, apply the Imaginary Frequency Protocol.

2. **TD-DFT vertical excitation energy calculation of T_1 at the S_0-optimized geometry**

   - If de-excitations are detected, flag and discard the molecule.
   - If the vertical excitation energy is below 1.7 eV, flag the molecule.

3. **TD-DFT vertical excitation energy calculation of S_1 at the S_0-optimized geometry**

   - If de-excitations are detected, flag and discard the molecule.
   - If the vertical excitation energy is below 1.7 eV, flag the molecule.

4. **DSCF optimization of T_1 (no frequency calculation)**

   - Start from the S_0-optimized geometry.
   - If the DSCF optimization converges within 200 cycles:
     - If the adiabatic excitation energy is smaller than 1.5 eV, flag the molecule.
     - Compare the T_1 and S_0 geometries using the connectivity matrix:
       * If the matrices are identical, proceed.
       * If differences are due to bond breaking, cyclization, or tautomerism, flag and discard the molecule.
     - Perform a single-point TD-DFT calculation for T_1 at its DSCF-optimized equilibrium geometry:
       * If the excitation energy with respect to S_0 at the T_1 equilibrium geometry is ≤ 0, or if de-excitations are detected, flag and discard the molecule.

- If the DSCF optimization does not converge within 200 cycles:
  - If the optimization fails to converge due to PCM-related issues, apply the PCM Error Protocol; otherwise:
  - Compare the T_1 geometry at the lowest energy found so far with the S_0 equilibrium geometry using the connectivity matrix:
    * If the matrices are identical, proceed.
    * If differences are due to bond breaking, cyclization, or tautomerism, flag and discard the molecule.
  - Perform a TD-DFT single-point calculation for the T_1 geometry corresponding to the lowest energy found so far:
    * If the excitation energy with respect to S_0 at the T_1 equilibrium geometry is ≤ 0, or if de-excitations are detected, flag and discard the molecule; otherwise, apply the restart procedure (steps a–c) described in Step 1.
    * If all restart attempts fail, flag and discard the molecule.

5. **TD-DFT optimization of S_1 (no frequency calculation)**

- Start from the T_1-optimized geometry.
- If the TD-DFT optimization converges within 200 cycles:
  - If the adiabatic excitation energy is below 1.7 eV, flag the molecule.
  - If the excitation energy with respect to S_0 at the S_1 equilibrium geometry is ≤ 0, or if de-excitations are detected, flag and discard the molecule.
  - Compare the S_1 and S_0 geometries using the connectivity matrix:
    * If the matrices are identical, proceed.
    * If differences are due to bond breaking, cyclization, or tautomerism, flag and discard the molecule.
- If the TD-DFT optimization does not converge within 200 cycles:

  - If the optimization fails to converge due to PCM-related issues, apply the PCM Error Protocol; otherwise:
  - Compare the S_1 geometry at the lowest energy found so far with the S_0 equilibrium geometry using the connectivity matrix:
    * If the matrices are identical, proceed.
    * If differences are due to bond breaking, cyclization, or tautomerism, flag and discard the molecule.
  - If de-excitations are detected at the last TD-DFT cycle, flag and discard the molecule; otherwise, apply the restart procedure (steps a–c) described in Step 1.
  - If all restart attempts fail, flag and discard the molecule.

6. **Frequency calculations for T_1 and S_1 (non-discarded molecules only)**
   - If imaginary frequencies are detected, apply the Imaginary Frequency Protocol.

Imaginary Frequency Protocol

- Identify the normal mode Q_1 corresponding to the largest-magnitude imaginary frequency (Gaussian reports it as negative).
- Displace the geometry by +0.5 along Q_1 (Q_1 ranges between 0 and 1, representing a normalized vibrational mode with unit total displacement).
- Restart the optimization with `opt(maxcycle=200,maxstep=5)`.

PCM Error Protocol

- If the optimization fails due to PCM errors, restart from the geometry with the lowest energy found so far.

- Increase the van der Waals radii scaling factor α from 1.1 (the Gaussian default) to 1.3 to enlarge the cavity.
- Optimize with `opt=loose`.
- After optimizing with $\alpha = 1.3$ and `opt=loose`, continue by optimizing the same geometry using $\alpha = 1.1$ and `opt=tight`.
